# Therapeutic Promises of Medicinal Plants in Bangladesh and Their Bioactive Compounds against Ulcers and Inflammatory Diseases

**DOI:** 10.3390/plants10071348

**Published:** 2021-07-01

**Authors:** Sheikh Rashel Ahmed, Muhammad Fazle Rabbee, Anindita Roy, Rocky Chowdhury, Anik Banik, Khadizatul Kubra, Mohammed Mehadi Hassan Chowdhury, Kwang-Hyun Baek

**Affiliations:** 1 Department of Plant and Environmental Biotechnology, Faculty of Biotechnology and Genetic Engineering, Sylhet Agricultural University, Sylhet 3100, Bangladesh; rashel.peb@sau.ac.bd (S.R.A.); krisarpita@gmail.com (A.R.); anikbanik.bgesau@gmail.com (A.B.); 2 Department of Biotechnology, Yeungnam University, Gyeongsan 38541, Korea; rabbi_biotech@ynu.ac.kr; 3 School of Medicine, Deakin University, 75 Pigdons Rd, Waurn Ponds, Melbourne, VIC 3216, Australia; rocky.mbcu@gmail.com; 4 Department of Biotechnology and Genetic Engineering, Faculty of Science, Noakhali Science and Technology University, Noakhali 3814, Bangladesh; khadiza@nstu.edu.bd; 5 Department of Microbiology, Faculty of Science, Noakhali Science and Technology University, Noakhali 3814, Bangladesh

**Keywords:** medicinal plants, anti-ulcer, inflammation, bioactive compounds, metabolites, therapeutic uses

## Abstract

When functioning properly, the stomach is the center of both physical and mental satisfaction. Gastrointestinal disorders, or malfunctioning of the stomach, due to infections caused by various biological entities and physiochemical abnormalities, are now widespread, with most of the diseases being inflammatory, which, depending on the position and degree of inflammation, have different names such as peptic or gastric ulcers, irritable bowel diseases, ulcerative colitis, and so on. While many synthetic drugs, such as non-steroidal anti-inflammatory drugs, are now extensively used to treat these diseases, their harmful and long-term side effects cannot be ignored. To treat these diseases safely and successfully, different potent medicinal plants and their active components are considered game-changers. In consideration of this, the present review aimed to reveal a general and comprehensive updated overview of the anti-ulcer and anti-inflammatory activities of medicinal plants. To emphasize the efficacy of the medicinal plants, various bioactive compounds from the plant extract, their experimental animal models, and clinical trials are depicted.

## 1. Introduction

For thousands of years, humans have been using medicinal plants, also referred to as healthy herbs, and have a long history of use in primitive medicines [1]. Traditional medicine prepared from these plants is still recognized as a preferred method in the health care system in many parts of the world because of its usefulness and affordability in the treatment of diseases [2]. The history of the Sumerian civilization first provided the instances of hundreds of medicinal herbs (e.g., opium), which were listed on clay tablets. Moreover, in 1550 B.C., the Egyptian Ebers Papyrus (an ancient medical document) depicted more than 882 herbal remedies of illness and injuries [3]. For many centuries, miscellaneous bioactive compounds emerging from the medicinal arbor have been used as a precursor to treating various diseases [4]. Owing to the existence of diversity, these organic molecules may be used as models for different synthetic drugs [5]. In addition, medicinal plants have justified their abilities to deal with several life-threatening diseases like cancer, hepatitis, and acquired immunodeficiency syndrome (AIDS) [6,7,8]. Hence, drug researchers are investigating the abundant curing substances found in nature using ethnobotany and have succeeded in discovering hundreds of pioneer compounds and drugs like aspirin, digoxin, quinine, and opium. The four major branches of phytochemicals include alkaloids, glycosides, polyphenols, and terpenes [9]. The widespread use of bioactive compounds is now evident in various applied branches of science such as agrochemicals, geo-medicine, modern pharmacology, plant science, food industry, cosmetics, and nano-bioscience [10].

In developing and underdeveloped societies, plant medicines are preferred over modern synthetic medicines due to their easy availability and affordability [11]. Moreover, the demand for plant-originated products is growing throughout the world. More than 85% of people in Asia, Africa, Latin America, the Middle East, and approximately 100 million people in the European Union rely on traditional herbal medicine for health issues. Approximately 90% of people in certain countries still practice in, and use, plant-based medicines [12]. Although the regulations of these conventional medicines are very limited in many countries of the world, the World Health Organization (WHO) has developed a web-based network to ensure its safe and rational use [13]. Due to the role of medicinal plants in the development of powerful therapeutic agents, over 1.5 million practitioners of the traditional medicinal system are using medicinal plants in preventive, promotional, and curative applications [14]. In Europe, it is estimated that the demand for the phytochemical and plant extract-based market has grown from $833.7 million in 2014 to $1.25 billion in 2019, which also indicates the current situation of plant-based consumers’ health awareness in the country [15].

Ulcers and different inflammatory diseases of the gastrointestinal tract are very much in need of effective therapeutic methods, since many individuals, regardless of age and gender, are still victims of these inflammatory diseases and remain under continuous medication without any hope of permanent recovery. Many chemically synthesized drugs are now being used to treat ulcers; however, they leave a range of side effects in the long run. For example, non-steroidal anti-inflammatory drugs (NSAIDs) are widely used in the treatment of inflammatory diseases, which may raise the risk of blood clotting that results in heart attacks and strokes. Therefore, the search for plant-mediated drugs is intended to counter these harmful diseases [16,17,18]. More specifically, anti-inflammatory drugs extracted from plants are being considered [19]. In addition, the application of herbal therapy to treat inflammatory bowel disease (IBD) is preferred worldwide because of its effectiveness and safety, although the relevant clinical trials are relatively limited to date [20]. Drugs such as amino-salicylates, corticosteroids, and immune-modulators are used in the treatment of ulcerative colitis, however, medicinal plants may be an effective and safe alternative to such medications [21]. 

The encouraging and ensuring health benefits of various clinical trials increased the acceptance of plant medicines among common people. Further research and investigations into the diverse active components of herbs and their clinical roles will illuminate and instigate the therapeutic use of plant-based medicines in the future [22]. This review focuses on supporting the therapeutic use of medicinal plants available in Bangladesh in the treatment of various inflammatory diseases of the gastrointestinal tract. Here, we have considered and reviewed the published articles using the keywords: medicinal plants, bioactive compounds, ulcers, inflammation, anti-ulcers, and anti-inflammatory diseases since 2000, from different databases like PubMed (https://pubmed.ncbi.nlm.nih.gov/) (accessed on 21 May 2021), Scopus Database (https://scopus.com) (accessed on 21 May 2021), and Google Scholar (https://scholar.google.com/) (accessed on 21 May 2021). The research findings of relevant medicinal plants, native or cultivated, in Bangladesh were taken into consideration. 

## 2. Inflammatory Diseases and Ulcers

Inflammation is a natural reaction in the defense of tissues against various injuries caused by physical stress combined with harmful chemicals or microbes [23]. Inflammation tends to be one of the prime causes for the occurrence of various diseases such as cancer, obesity, cardiovascular disease, rheumatoid arthritis, osteoporosis, asthma, IBD, central nervous system (CNS) depression, diabetes, and Parkinson disease [24]. Inflammation can be triggered by many stimuli including pathogens or cytokines (i.e., interleukin-6 or IL-6, tumor necrosis factor-alpha or TNF-α, neutrophils, and monocytes). These stimulating agents can be differentiated into macrophages, which are subsequently attracted to injured tissue sites by chemotaxis and intensify the inflammatory reactions to the damaged areas as well as initiate phagocytosis [25]. Inflammation can be characterized by swelling, joint pain, and redness. Inflammation leads to several chronic diseases including arthritis, autoimmune disease, coeliac disease, colitis, and asthma, which are often associated with the increased risk of development of cardiovascular diseases, diabetes, cancer, and osteoporosis [26]. Phytochemicals derived from medicinal plants can be used in the alleviation of inflammatory reactions by inhibiting different forms of enzymes e.g., lipoxygenase (LOX), cyclooxygenase (COX), phospholipase A2, and proteins (e.g., inhibition of the pro-inflammatory cytokines) [23]. In medical science, Crohn’s disease, and ulcerative colitis (UC) are two types of IBD that cause inflammation in the entire gastrointestinal tract and colonic mucosa, raising the risk of colon cancer as well [27].

An ulcer is the condition of corrosion in the linings of the stomach and the duodenum. Thus, ulcers in the gastrointestinal tract are subdivided into ulcerative colitis (lower) and peptic ulcers (upper) depending on the location of the infection [28,29]. Peptic ulcers, also known as gastric and duodenal ulcers, can be characterized as submucosal damage of the digestive tract caused by the disruption of the balance between the hostile factors (i.e., gastric acid, *Helicobacter pylori*, and anti-inflammatory drugs) and protective factors (i.e., mucus, bicarbonate, prostaglandins, and blood flow towards the mucosa) [30]. The common symptoms of peptic ulcers include a burning sensation and pain in the middle or upper stomach, bloating, heartburn, nausea or vomiting, and weight loss [31]. Excessive consumption of alcohol, smoking, chewing tobacco, serious illness, and the intake of NSAIDs increase the risk of ulcer development [28,32]. Gastric ulcers and duodenal ulcers, which are more prevalent in the Eastern and Western countries, cause morbidity and mortality worldwide [33], and *H*. *pylori* are considered as one of the most important factors in the development of this disease [34].

## 3. Plant Mediated Treatment of Ulcer and Inflammatory Diseases

Medicinal plants are the blessings of nature that humans have been using since prehistoric times as the precursor of most drugs. The treatment of ulcers and IBD is facilitated by the intensive use of many medicinal plants [35]. In addition, in recent years, numerous studies on plant extracts have substantially demonstrated anti-ulcer and anti-inflammatory activities in both in vitro and in vivo models.

Drugs derived from medicinal plants are effective against inflammation of the digestive tract primarily in two ways, either by reducing acid and pepsin secretion or by assisting the cytoprotection via mucosal defense factors [36]. The mode of action of these drugs differs according to their function. These medications maintain a balance between several aggressive factors (i.e., pepsin, acid, bile salts, and *H. pylori*) and defensive factors (i.e., cellular mucus, mucin secretion, mucosal blood flow, bicarbonate secretion, and cell turnover) [36,37]. For instance, the methanol extract of drumstick leaves, and flower buds inhibited aspirin-induced gastric lesion formation in rats [38]. It has been reported that several compounds, such as cavidine, chelerythrine, quercetin, hesperidin, α-pinene, and garcinol, present in medicinal plants were used against ulcer diseases [39]. Additionally, prominent anti-inflammatory compounds such as resveratrol, colchicine, epigallocatechin-3-gallate (EGCG) capsaicin, phytosterols, saponins, and curcumin derived from plants were used to treat inflammatory diseases [40,41].

In addition to the use of traditional medicines such as NSAIDs, the use of proton pump inhibitors (e.g., pantoprazole, omeprazole, lansoprazole, and rabeprazole), histamine receptor blockers (e.g., famotidine and ranitidine), synthetic prostaglandin E1 (e.g., misoprostol), antacids (e.g., aluminum hydroxide and magnesium trisilicate combination), corticosteroids (e.g., dexamethasone), immune-suppressants, immune-modulators (e.g., azathioprine and 6-mercapto-purine), antibiotics (e.g., clarithromycin and metronidazole), and biologic agents (e.g., TNF-α) for the treatment of inflammatory diseases is in practice on a large scale and has significant adverse and undesirable side effects including gastrointestinal and hepatic toxicity, renal and cardiovascular malfunctions, and hematologic effects such as hemophilia and thrombocytopenia [32,42,43,44,45]. As a result, the use of natural products such as plants and herbal derivatives is increasingly growing among ulcerated and inflammatory patients having minimal or no side effects as they are the products of nature. Different types of plants which are currently considered to be effective to treat ulcers and inflammatory diseases are discussed below:

### 3.1. Aegle marmelos

*A. marmelos* of the *Rutaceae* family is known most often as the ‘bael’ tree in subcontinent regions and as the ‘wood apple’ in other parts of the world [46,47]. All parts of this plant (leaf, root, seed, bark, and fruit) contain medicinal properties and have many active chemical constituents, such as flavonoids (e.g., flavone and rutin) [48,49], tannins (e.g., skimmianine) [50], phenylpropanoids (e.g., lignans and phenylpropenes) [51], saponins, luvangetin, coumarins (e.g., xanthotoxol, marmelosin, and scoparone) [52], carbohydrates (e.g., galactose, L-rahamanos, etc.), terpenoids, essential oils (e.g., phellandrene, limonene, ocimene, and pinene) [53], ester, and alkaloids (e.g., skimmiarepin and cinnamamide) [54,55]. The ripe and unripe fruits of this plant have historically been used in the treatment of ulcers, chronic diarrhea, dysentery, rectal inflammation, and certain cancers [56]. Additionally, leaves from *A. marmelos* soaked in water overnight can control peptic ulcers [37] and root decoction is used to treat fevers and colds [56]. 

**Figure 1:**
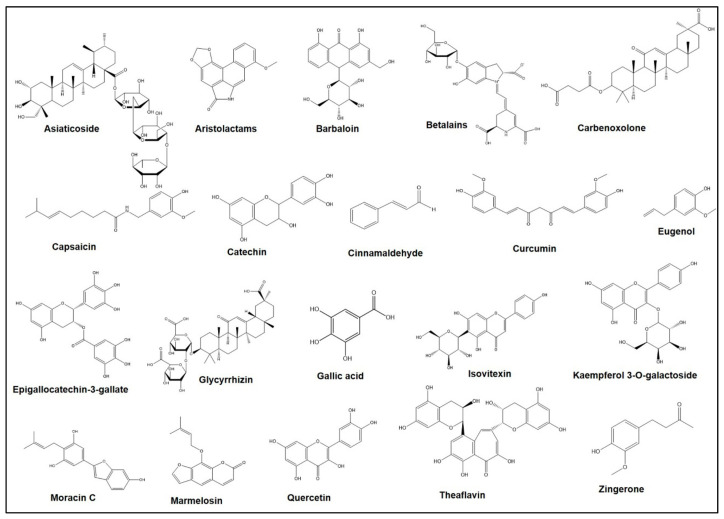
Chemical structures of different bioactive compounds derived from the medicinal plants.

Extensive experimental studies have demonstrated that *A. marmelos* may prevent ulcers and inflammation effectively. An aqueous extract of *A. marmelos* resulted in decreasing the ulcer score and ulcer index in rats’ stomachs, which was successfully achieved after the administration of 400 mg/kg of an aqueous extract from *A. marmelos* [57]. In other studies, ethanol, and an aqueous extract from *A. marmelos*, provided 56.33% and 37.2% protection of ulcers in vivo and the ethanol-induced gastric ulcer model, respectively, compared to standard drugs such as omeprazole (50.4% protection) (20 mg/kg body weight) [57,58]. Ramakrishna et al. investigated the effect of the oral administration of methanol extract from unripe *A. marmelos* in rats against *H. pylori* LPS-induced gastric ulcers which showed that this extract (500 mg/kg dose) caused a 93.98% reduction in the gastric ulcers [59]. This improvement in gastric ulcers was due to the inhibition of parameters of gastric secretion, including free and total acidity, pepsin concentration, acid release, and the volume of gastric juice [59]. Similarly, from another study, the extract from the unripe fruit of *A. marmelos* at a dose of 50 and 100 mg/kg (intraperitoneal injection) also exhibited gastro-protective action against ethanol-induced gastric mucosal damage [60]. These findings suggest that the *A. marmelos* and the active compounds in it trigger the prostaglandin-independent pathway as a gastro-protective mechanism. Bioactive compounds exerting the gastro-protective properties were not specifically investigated, but the major compounds like eugenol, luvangetin, marmin, marmelosin, mucilage, aegelin, dictamine, and auroptene might trigger gastro-protective activities [59,61,62]. Marmelosin (molecular formula C_16_H_14_O_4_) (Figure 1) showed the ability to fight against cellular and DNA damage which are vital events that trigger inflammation. Moreover, the downregulation of the pro-inflammatory marker (TNF-α) and the transcription factor (NFĸB) by marmelosin inhibits chronic inflammation in cancer [63]. Eugenol (molecular formula C_10_H_12_O_2_) (Figure 1), another major bioactive compound of *A. marmelos*, can help to cure ulcers as it is significantly effective in reducing myeloperoxidase (MPO) activity, which is responsible for the neutrophilic infiltration during ulcer formation [64]. Moreover, eugenol inhibits the production of superoxide anion by neutrophils which results in the reduction of MPO substrates, hydrogen peroxides, and ultimately triggers the decline of MPO activity in neutrophils [65].

### 3.2. Aloe vera

*A. vera* from *Liliaceae* is known as ‘ghritkumari’ in Bangladesh and India, and ‘*Aloe vera*’ worldwide [66]. The leaves, mainly, of *A. vera* have great medicinal importance as they have active chemical compounds such as saponins, essential amino acids (e.g., cysteine, alanin, arginine, and histidine); anthraquinones (e.g., aloetic acid, aloin A and B (or collectively known as barbaloin), anthracine, anthranon, emodin, etc.); enzymes (e.g., alkaline phosphotase, amylase, catalase, cellulase, cyclooxidase, and lipase); hormones (e.g., auxin and gibberllins); chromones, lignin, minerals, salicylic acid, sterols, carbohydrates (e.g., lignins and sugars); dietary fibers, protein, organic acids, lipids, and vitamins [67]. For the treatment of ulcers, rural people take the inner gel layer of fleshy leaves orally with water [68]. This plant has great potential for curing and preventing gastric ulcers by stimulating its anti-inflammatory and healing function and by regulating the mucus and gastric secretion [69]. *A. vera* can successfully treat various illnesses and conditions including duodenal ulcers, peptic ulcers, mouth ulcers, and sore throats [70]. *A. vera*, coupled with silver nanoparticles, was effective for ulcer healing by their anti-inflammatory enhanced re-epithelialization as well as fibroblast activation effects [71]. *A. vera* extract pre-treated animals (dose of 200 mg/kg bwt for five days) had reduced signs of mucosal injury relative to untreated controls, although the incidence was not as high as in omeprazole-treated rats [72]. On the contrary, *A. vera* extract pre-treatment was ineffective against gastric lesion formation [73]. Furthermore, the extract of *A. vera* has been shown to cause dose-dependent amelioration in the severity, as well as incidence, of acetic acid-induced gastric lesions when used as a preventive measure in rats [74]. Aloin A and B (molecular formula: C_21_H_22_O_9_), collectively known as barbaloin (Figure 1), is considered one of the prominent constituents isolated from *A. vera* which showed potency to treat ulcers and inflammatory diseases [75]. To treat ulcerative colitis, barbaloin effectively increases the mRNA expression of IL-4 and IL-10 in tissues and simultaneously decreases the expression of IFN-γ, IL-6, IL-1β, and TNF-α [76]. Furthermore, barbaloin can prevent the ulcer-mediated myosin light chain kinase (MLCK) signaling pathway by activating the 5’ adenosine monophosphate-activated protein kinase (AMPK) signaling pathway.

### 3.3. Alpinia nigra and A. species

The decocted roots of *A. nigra* (*Zingiberaceae* family), locally known as ‘bhulchengi’ or ‘khetranga’, are used as a treatment of gastric ulcers [77]. Natural bioactive compounds as well as crude hydroalcoholic fractions isolated from the *Alpinia* spp. exhibits anti-inflammatory and analgesic activities. Hydroalcoholic and aqueous extracts from the rhizomes and leaves of different *Alpinia* spp. have bioactive components with anti-nociceptive and anti-allergic characteristics [78]. Recently, platelet-activating factor antagonists, named diarylheptanoids, have been reported from *Alpinia officinarum* rhizome extract [79], which also exhibited analgesic actions with 80% ethanoic extract [80]. Fruit extract of *A. nigra* at a concentration of 250 mg/kg and 500 mg/kg exhibited dose-dependent potential against inflammatory paw edema induced by carrageenan. These healing properties may be due to the existence of a large amount of terpenoid, tannins, alkaloids, and flavonoids [81]. Diarylheptanoid, flavonoids, and some volatile oils (e.g., cineole alpha-pinene, sesquiterpene, linalool, lactones) which are the major bioactive components isolated from *Alpinia* species have several biological activities including anti-oxidant and anti-cancerous due to the presence of functional aldehyde (–CHO) and keto (C=O) groups in their structure [82]. 

### 3.4. Annona squamosa 

*A. squamosa* of the *Annonaceae* family is generally referred to as the ‘custard apple’ [83]. Based on the phytochemical analysis, alkaloids, total phenolic compounds (TPCs), including flavonoids, saponins, tannins, and phenols, are most abundant in *Annona squamosa*. It has been reported that these phenolic compounds have regulatory effects on several physiological and biochemical processes (e.g., signal transduction pathways, cell proliferation, enzymatic activity, and cellular redox potential) to cure chronic diseases [84]. The bark, leaves, stems, roots, seeds, and fruits of *A. squamoza* contain active alkaloid compounds (e.g., annosqualine, dihydrosinapoyltyramine, and liriodenin) [85]; sugar (e.g., quercetin-3-*O*-glucosid) [86]; phenolics, flavonoids, and tocopherol [87]; acetogenins (e.g., squamostatins, squamoxinone, mosinone, and bullacin) [88], essential oils rich in ent-kaurane type diterpenoids and sequeterpenes (e.g., β-caryophyllene, and β-cedrene) [88].

*A. squamoza* had anti-tumor, anti-oxidant, anti-diabetic, anti-lipidimic, anti-ulcer, and anti-inflammatory properties due to its cyclic peptide contents [89]. In this phytochemical and in vivo pharmacological research, twelve known compounds were isolated and, among them, 1-(4-β-d-glucopyranosyloxyphenyl)-2-(β-d-glucopyranosyloxy)-ethane was isolated from natural sources for the first time. It has been shown that some isolated compounds provided gastroprotection through the inhibition of H^+^ K^+^-ATPase (proton pump) activity and the boosting of the mucosal defense mechanism simultaneously. *A. squamosa* leaves have been shown to have substantial analgesic as well as anti-inflammatory activities compared to synthetic drugs such as aceclofenac and pentozocine, indicating a mixed central as well as a peripheral mechanism for its effectiveness [90]. Additionally, the literature suggested that leaf extract of *A. squamosa* has both thyroid inhibitory and anti-peroxidative properties [91], which are also beneficial during the proper functioning of lipid and carbohydrate metabolism [92].

### 3.5. Anthocephalus cadamba 

Various parts of *A. cadamba* (Family: *Rubiaceae*) are used in the treatment of numerous conventional health problems, including mouth ulcers, stomatitis, subdermal inflammatory deposits, and gastric disturbances [93]. The anti-ulcer action of methanolic and aqueous extracts of *A. cadamba* bark and leaves were examined in both aspirin-induced and pylorus-ligation ulcer models [94]. At oral doses of 200 and 400 mg/kg, both extracts demonstrated the substantial inhibition of gastric lesion in aspirin-induced gastric ulcers and pylorus ligation-induced ulcers. At the same time, the extracts also exhibited a significant decrease in gastric volume, free acidity, pH, total acidity, ulcer inhibition, as well as ulcer index [94]. The gastro-protective efficacy of *A. cadamba* was investigated in an in vivo mice study showing the ethanolic extract of *A. cadamba* at two doses of 250 and 500 mg/kg substantially decreased ulceration caused by both HCl and ethanol-dependent doses [95]. Similarly, in another report, the defatted aqueous extract of the leaves of this plant showed substantial analgesic and anti-inflammatory activities at different doses at 50, 100, 300, and 500 mg/kg [96,97]. In addition, the methanol extract of the *A. cadamba* bark has also been successfully assessed for antipyretic, analgesic, and anti-inflammatory actions by some researchers [98,99].

### 3.6. Aristolochia indica 

This plant (family: *Aristolochiaceae*) has been applied against both ulcers and anti-inflammatory diseases and so on [100]. Aristolochic acids and esters, aristolactams, terpenoids, and flavonoids are the major groups of phytoconstituents that are common in all species of *Aristolochia* including *Aristolochia indica*. Among them, aristolactams (molecular formula: C_17_H_11_NO_4_) (Figure 1) are synthesized as biogenetic intermediates which are normally supposed to be generated from the reduction products of aristolochic acids through the cyclization condensation reaction. From the *Aristolochia* species, twelve aristolactams have been reported, and among them, there were also six compounds having the 3,4-methylenedioxy substitution groups [101]. Anti-inflammatory effects of the isolated compounds aristolactam I and hinokinin from *A. indica* have been reported against TNF-α and IL-6 respectively [102]. (-) Hinokinin exhibited its anti-inflammatory effects through the nuclear factor kappa-light-chain-enhancer of the activated B cells (NF-κB) dependent mechanism whereas aristolactam I followed the NF-κB independent mechanism. The ethanol extract of the root bark of *A. indica* successfully inhibited inflammatory activity induced by a hyposensitive agent, named “compound 48/80” (a condensation product of N-methoxyphenethylamine and formaldehyde), which has an almost identical inhibitory pattern of synthetic anti-inflammatory drugs such as ketotifen fumarate [103]. 

### 3.7. Artocarpus heterophyllus

*A. heterophyllus* Lam. from the *Moraceae* family is locally called ‘kanthal’. The leaf ash is taken orally to treat ulcers and young leaves in combination with roots are often beneficial for skin problems, respiratory diseases, and diarrhea (oral medication) [104]. The methanolic extract of *A. heterophyllus* at a concentration of 500 mg/kg inhibited the indomethacin-induced gastric ulceration, decreased gastric acid concentration, and increased gastric pH concentration [105]. In another experiment, the natural phenolic compound Moracin-C (molecular formula: C_19_H_18_O_4_) (Figure 1) was isolated from *A. heterophyllus*, and has been proven to have considerable anti-inflammatory effects by blocking the release of lipopolysaccharide (LPS), activated nitric oxide (NO), and reactive oxygen species (ROS) without showing detectable cytotoxicity [106]. Moreover, the compound Moracin-C significantly decreased LPS-stimulated mRNA up-regulation and protein expression of inducible cyclooxygenase-2 (COX-2), nitric oxide synthase (iNOS), and several pro-inflammatory cytokines (e.g., IL-1, IL-6, and TNF-α). The anti-inflammatory activity of Moracin-C was linked with the activation of the mitogen-activated protein kinases (e.g., p38, ERK, and JNK) as well as NF-κB pathways by decreasing the nuclear translocation of NF-κB p65 subunit as observed by confocal microscopy and nuclear separation experiment [106].

### 3.8. Asparagus racemosus

The *A. racemosus* of the *Liliaceae* family is locally known as ‘shatamuli’ in Bangladesh and ‘climbing asparagus’ all over the world [107,108]. Different parts of *A. racemosus* contain different bioactive compounds such as steroidal glycosides, steroidal saponins (e.g., shatavarin I, shatavarin IV); flavonoids (e.g., 8-methoxy-5, 6, 4′- trihydroxyisoflavone); alkaloids (e.g., asparagamine A); asparagamine, racemosol, beta-sitosterol, stigmasterol, genistein, and daidzein [108,109,110,111]. The plant, particularly its root, has been shown to have anti-ulcerogenic, adaptogenic, anti-oxidant, and anti-dyspepsia activities [112]. The in vivo research indicated the inhibitory effect of *A. racemosus* on gastric hydrochloric acid secretion that protects gastric mucosal damage [113]. In addition, the reduction in ulcer index using the raw extract of *A. racemosus* (100 mg/kg/day orally) was comparable to the antiulcer drug ranitidine (30 mg/kg/day orally). The bioactive compound, shatavarin (steroidal saponin), isolated from *A. racemosus*, acts spontaneously in the treatment of ulcers in a chemically-induced ulcer model by increasing the mucin secretion inside the gastrointestinal tract [114]. 

### 3.9. Beta vulgaris

*B. vulgaris* of the *Chenopodiaceae* family is generally referred to as ‘beetroot’ or ‘sugar beet’, widely used as a vegetable or as a salad [115]. The root decoction along with a small amount of vinegar is traditionally used for the treatment of ulcers and sores. The alcoholic root extract of *B. vulgaris* at a concentration of 200–400 mg/kg significantly decreased the ulcer index, ulcer score, total acidity, and maintains normal mucosa in pylorus ligation and ethanol-induced ulcer in rat models [116,117]. The active constituents in *B. vulgaris* are polyphenols, tannins, alkaloids, vitamins (e.g., C, B3, B6, B9), carotenoids, flavonoids, betacyanins, betaxanthins, betanin, and saponins, most likely have inhibitory effects on gastric mucosal injury [116,118,119]. Betalains (molecular formula: C_24_H_26_N_2_O_13_) (Figure 1), nitrogenous water-soluble compounds, are abundant in *B. vulgaris* which have strong therapeutic activity against inflammatory diseases. This phenolic chromoalkaloid synthesized from the amino acid tyrosine is used as food additives due to its high solubility and lack of toxicity [120]. Pre-treatment with petroleum ether extract of *B. vulgaris* L. gave significant defense against aspirin-induced gastric ulcers [121]. The anti-ulcer effects are likely due to the existence of steroids or phenols in the extract. Moreover, there are significant anti-inflammatory properties in the aqueous extract of *B. vulgaris* which was also investigated against the carrageenan-induced edema in rats [122]. 

### 3.10. Bombax ceiba

The plant *B. ceiba* is under the family *Bombacaceae* and has several medicinal properties against inflammatory diseases, such as cancer, dysentery, ulcer, and microbial infections [123]. Bark, latex, leaf, flower, seed, stem, rhizomes, thorn, stem bark, fruit, and heartwood are the medicinally active parts of *B. ceiba* which contain common flavonoids (e.g., isovitexin (molecular formula: C_21_H_20_O_10_) (Figure 1), kaempferol 3-*O*-galactoside (molecular formula: C_21_H_20_O_11_) (Figure 1), gallic acid (molecular formula: C_7_H_6_O_5_) (Figure 1)), quercetin, lupeol, tannic acid, sesquiterpenoids, naphthol, naphthoquinones, polysaccharides, anthocyanins, shamimin, lupeol, and alkaloids [123,124]. The young root paste of this plant is traditionally used in the treatment of gynecological disorders, constipation, diarrhea, piles, dysentery, wounds, ulcer, inflammation, and urinary diseases [124,125]. Crude plant extracts demonstrated strong analgesic efficacy in acetic acid-induced writhing as well as hot plate tests in mice [126]. Using naloxone, *B. ceiba* extract-induced analgesia was independent of the opioid receptor; where, mangiferin exhibited significant interaction with the receptor at a peripheral site, with a minor contribution at the neuronal stage [126]. This plant is known to have large amounts of gallic and tannic acids that act as astringents precipitating proteins that help to regenerate the damaged epithelial mucosal lining of the ulcerated lesion [127,128]. Similarly, *B. ceiba* effectively decreased the ulcer index after administration of flower extract (300 mg/kg/day) for seven days, and suppressed gastric inflammation by inhibiting TNF-α and interleukin 1β (IL-1β) [129].

### 3.11. Caesalpinia pulcherrima 

*C. pulcherrima* of the family *Fabaceae* or *Leguminosae* is locally known as ‘krishnachura’ in Bangladesh and as ‘peacock flower’ worldwide [130]. Various medicinally active vegetative sections of this plant including young leaves, bark, fruit, seed, stem, flower, and whole plant are considered as a storehouse of different bioactive compounds such as sterols (e.g., β-sitosterol), flavonoids (e.g., flavones, isoflavones, flavanols chalcones, rotenoids), glycosides, organic compounds (e.g., α-phellandrene), essential oils (e.g., β-caryophyllene, γ-Terpinene), and carotenoids (e.g., lutein, zeaxanthin), etc. [130,131,132]. Traditionally, the extracts of this plant have been used to treat various diseases such as malaria, diarrhea, dysentery, fungal infections, respiratory diseases, inflammatory diseases, and microbial diseases [132]. The gastroprotective ability of galactomannan extracted from the seeds of *C. pulcherrima* L. (GM-CP) was assessed in indomethacin induced acute gastritis model, which showed that GM-CP (10 mg/kg dose) decreased the severity of macroscopic lesion as well as the loss of superficial cells through ameliorating inflammatory symptoms including neutrophil infiltration, production of TNF-α, thiobarbituric acid, reactive species migration, and adhesion of mesenteric leukocytes [133]. Sharma and Rajani have assessed the anti-inflammatory and anti-ulcer efficacy of *C. pulcherrima* in indomethacin induced cotton pellet granuloma and both aspirin and pylorus-ligation-induced ulcer models respectively [134]. 

### 3.12. Calendula officinalis 

*C. officinalis* L. (family: *Asteraceae*) is popular with the name ‘gada ful’ to Bangladeshi people. The petals are orally administered in the treatment of stomach pain, inflammation, and ulcers [135]. The extract of *C. officinalis* had both antacid property and gastroprotective capacity. Significant gastro-curative efficacy of the extract was investigated against absolute ethanol and indomethacin induced ulcerative lesion in rats [136]. Another study also confirmed the presence of gastroprotective properties in the plant extract of *C. officinalis* resulting in the substantial inhibition of the ulcer development caused by chemical and physical agents with 87.15% utmost therapeutic efficiency (450 mg/kg bwt) in cold-resistant and stress-induced ulcers [137]. The findings from lipid peroxidation and enzyme assay clearly exhibited the antioxidant effect of the whole plant of *C. officinalis* on the ulcer condition. Likewise, the ethanolic extract of *C. officinalis* in mice showed substantial anti-inflammatory properties against carrageenan as well as dextran–induced paw edema [138]. The extracts substantially inhibited enhanced extents of pro-inflammatory cytokines (e.g., IL-6, IL-1β, TNF-α, IFN-γ) and acute-phase protein, C-reactive protein (CRP), in mice generated by LPS injection. The extract also inhibited LPS-induced COX-2 levels in the spleen of mice. The findings demonstrated that potential anti-inflammatory activities of *C. officinalis* extract may be mediated by inhibiting pro-inflammatory cytokines as well as COX-2, and subsequent prostaglandin synthesis [138]. 

### 3.13. Calotropis procera

*C. procera* of the *Asclepiadaecae* family is popularly termed ‘akanda’ in Bangladesh and ‘milkweed’ worldwide [139]. The leaf, flower, flower bud, latex, root bark, and root of this plant contain different bioactive compounds such as triterpenoids, lupeol, flavonoids, flavanols, glycosides, resins, cardenolides, mudarine, anthocyanins, α-amyrin, β-amyrin, calactin, calotropin, and β-sitosterol. Bioactive compounds from *C. procera* show protective properties against different diseases [139,140]. Traditionally, the latex is used in reducing toothache [141], in the treatment of vertigo, paralysis, hair loss, baldness, and rheumatoid/joint swellings. In addition, the leaves have therapeutic values to reduce swelling as well as to treat joint pain. The bark is used in the treatment of eczema, leprosy, and elephantiasis treatment [142].

Different parts of *C. procera* show anti-inflammatory and gastroprotective properties. The leaves and bark of *C. procera* have curative properties for ulcers and stomach aches. The anti-ulcer efficacy of *C. procera* extract was determined in white albino rats [143]. The extract significantly reduced the ulcer index, ulcer lesion score, leukocyte infiltration in the gastric epithelial lining, and ameliorated the congestion and necrosis. The anti-ulcer property of the plant was mainly due to the presence of different phyto-constituents like polyphenols, flavonoid and lignin glycoside triterpenoids, tannins, and steroids [144]. In the treatment of ulcerative colitis, colitic rats treated with the methanol extract of dried latex of *C. procera* (MeDL), demonstrated a significant reduction of colonic mucosal damage. This reduction resulted from the inhibition of oxidative stress and pro-inflammatory signaling proposes *C. procera* as a promising therapeutic plant to treat inflammatory conditions in the colon [145]. From this study, it was proposed that targeting oxidative stress and NFκB (p65) regulated pro-inflammatory signaling could be a potential approach for providing protection in the treatment of colitis. 

### 3.14. Camellia sinensis

*C. sinensis* of the *Theaceae* family is locally termed as ‘cha’ in Bangladesh and as ‘black tea’ worldwide [146]. The leaves, stems, and twigs contain different active constituents like flavonoids (e.g., thearubigins, theaflavins, and catechins), vitamins amino acids, β-carotene, chlorogenic acids, volatile compounds carbohydrates, phenolic acids (e.g., gallic acid, caffeic acid, and cauramic acid), proteins, lipids, and fluoride [147,148]. These components help in the treatment of different diseases with their different protective properties [146]. This plant is traditionally used in the treatment of flatulence, digestion, vomiting, diarrhea, maintaining body temperature, blood sugar, and in the alleviation of stomach discomfort [149,150]. Heteropolysaccharides extracted from *C. sinensis* exerted gastroprotective properties by reducing ethanol-induced gastric lesions [151]. Moreover, it was also found effective in gastroprotection by gastric mucus maintenance and decreased glutathione levels [151]. 

Theaflavin (molecular formula: C_29_H_24_O_12_) (Figure 1), a major active component of black tea, showed the potency for the healing of indomethacin-mediated gastric ulcers by the synthesis of PGE2, revealing antioxidative aspects and intensification of mucin secretion [152]. This polyphelnolic compound also triggers the suppression of several inflammatory modulators in the ulcer margin and induces iNOS modulation for gastric ulcer healing [153]. Catechin (molecular formula: C_15_H_14_O_6_) (Figure 1), especially epigallocatechin-3-gallate (EGCG) (molecular formula: C_22_H_18_O_11_) (Figure 1), is the major bioactive polyphenol compound of *C. sinensis* and showed strong antagonistic activity against inflammatory respiratory infections in humans caused by *Stenotrophomonas maltophilia* [154]. This compound inhibits the enzyme dihydrofolate reductase of *S. maltophilia*, which is considered an attractive target for the development of antibacterial molecules [155]. Green tea catechins inhibited TNF synthesis in a murine model of inflammatory arthritis and reduced inflammation as well as slowed down cartilage breakdown [156].

### 3.15. Capsicum annuum and C. frutescens

*C. annuum* and *C. frutescens* from the *Solanaceae* family are known as ’chili’, ‘pepper’ or simply “capsicum” worldwide, contains different active constituents like solasonine, capsacin, acyclic diterpene glycosides, and capsidiol [130,157]. The fruit is used locally as a spice, which shows potent anti-ulcer and antioxidant properties. It also helps prevent type-2 diabetes [130,157]. The fruit of *C. frutescens* was taken to treat gastric disorders and ulcers [158]. At doses of 300 and 600 mg/kg body weight, the aqueous extract of the chili pepper (*C. frutescens*) reduced the gastric ulcer length of aspirin-induced ulcer in experimental rats, which proved the curing properties of the extract of *C. frutescen* [159]. Capsaicin (molecular formula: C_18_H_27_NO_3_) (Figure 1) is the major bioactive component of these plants, exhibits anti-inflammatory characteristics [160,161]. This secondary metabolite of *Capsicum* spp. is also known as capsaicinoid due to their alkaloid nature. Researchers assessed the impact of capsaicin on the mucosa of the stomach, pro-inflammatory cytokines (e.g., TNF-α, IL-6, IL-1β), and COX-2 in gastric mucosa in two experimental models. Histopathological examinations coupled with molecular studies of stomach samples revealed a protective action of gastric mucosa along with a substantial reduction of pro-inflammatory cytokines as well as COX-2 in both experimental models [160].

### 3.16. Carica papaya

*C. papaya* of the Caricaceae family is commonly known as ‘papaya’ [162]. *C. papaya* contains diverse active constituents like enzymes (e.g., papain, chemopapain, chymopapain, peptidase, lysosome, and myrosine), proteins, fats (e.g., myristic, palmitic, stearic, and linoleic), carbohydrates (e.g., glucose, fructose, galactose, and xylitol), minerals, vitamins, volatile compounds, alkaloids (e.g., carpain, pseudocarpain, choline, and caproside), glycosides, and carotenoids [163,164]. The most common and significant constituent, papain, is a papaya proteinase I (cysteine protease) enzyme which has several therapeutic effects particularly in inflammation and gastrointestinal disorders. Traditionally, the decoctions of leaves and dried flowers were used as anti-anemic agents, blood purifiers, and in several diseases [164,165,166]. Fruits are used to treat impotence and ulcer. The crude latex decoction is used to treat anthelmintic, dyspepsia, burns pain, bleeding hemorrhoids, stomachic, and diarrhea [167].

### 3.17. Cinnamomum cassia

*C. cassia* of the *Lauraceae* family is commonly known as ‘daruchini’ in Bangladesh and as ‘cinnamon’ worldwide. The bark of cinnamon contains different active constituents such as terpenoids, phenylpropanoids, glycosides, cinnamaldehyde, cinnamic acid, cinnamate, and numerous essential oils (e.g., trans-cinnamaldehyde, cinnamyl acetate, eugenol, L-borneol), etc. [168]. The *C. cassia* plant is traditionally used as a spice and cures dental problems, prevents colon cancer, and acts as a coagulant to prevent bleeding [169]. It performs different anti-inflammatory activities [168,169,170]. Cinnamaldehyde (molecular formula: C_9_H_8_O) (Figure 1), the most abundant phytocomponents of *C. cassia*, was active as an anti-inflammatory agent in gastric inflammation. This aromatic aldehyde compound inhibited IL-8 secretion/expression and the nuclear factor kappa B (NF-κB) pathway to treat *Helicobacter pylori*-induced gastric inflammation [171].

### 3.18. Chromolaena odorata L.

*C. odorata*, which is commonly known as ‘germany lata’ in Bangladesh, is a member of the *Asteraceae* family. Several chemical constituents including flavonoids, alkaloids, triterpenes/steroids, monoterpenes, and phenolic acids can play significant roles in treating inflammatory and ulcer diseases [172]. The plant has been grown in bushy areas, forest zones, and roadsides. The leaf extract of this plant with salt has been used as an oral medicine in the tropical areas of Bangladesh to get rid of ulcers [173]. The ethanol-induced gastric lesion was successfully minimized by applying the extract of *C. odorata* L. Also, *C. odorata* extract was effective in arresting internal bleeding due to the presence of polyphenols [174].

### 3.19. Curcuma longa L.

*C. longa* L. of the *Zingiberaceae* family is locally known as ‘halud’ in Bangladesh and India and commonly termed as ‘turmeric’ worldwide [175]. The rhizome and leaves of *C. longa* contain many active constituents such as alkaloids, phenolic compounds (e.g., isoflavone, diarylheptanoids curcuminoids, diyrenphenate), alcohols, essential oils (e.g., monoterpenes, sesquiterpenes, diterpenes, and triterpenoids), ketones, ß-turmerone, α-turmerone, steroids (e.g., β-sitosterol), and aldehydes [176,177,178]. Traditionally, this plant has been used to cure dermatitis, cancer, leprosy, hemorrhoids, inflammation, and asthma, and shows hepato-protective activity [179,180]. Based on its antioxidant property, curcumin (molecular formula: C_21_H_20_O_6_) (Figure 1), also known as diferuloylmethane, an active component of *C. longa*, scavenges ROS and regulates matrix metallopeptidases (MMPs) activity to induce antiulcer activity [181,182]. Beside this, curcumin also exerted different pharmacological effects including anti-inflammatory activity triggered by suppression of PG synthesis [183,184]. It is reported that the substitution group on the methoxy position draws a vital contribution in the anti-inflammatory effects of curcumin [185]. In a transgenic mice model, it was shown that phytosomal curcumin exerted strong effects on the activation of anti-inflammatory PPARγ (peroxisome proliferator-activated receptor γ) as well as the inhibition of pro-inflammatory NF-κB, therefore, it could be used in the treatment of patients with chronic hepatitis B infection [186]. The application of the paste of the rhizome is carried out on injuries, strains, and wounds externally as the primary treatment. The leaves are also used in malaria and jaundice treatment and during pregnancy [187,188]. From the findings of phase-I clinical trials and toxicology studies conducted by Anand et al., it was concluded that curcumin is safe and tolerated even at very high doses (12 g/day) in humans [189]. A joint report from the World Health Organization (WHO) and the Food and Agriculture Organization (FAO) has recommended that a high intake of curcumin at a concentration of 0-1 mg/kg body weight per day had no adverse health effects on the human body [178].

### 3.20. Glycyrrhiza glabra

*G. glabra* (family: *Fabaceae*) has been reported to treat gastric ulcers, oral ulcers, as well as ulcerative colitis [190]. Although the powder form of *G. glabra* is commonly used to treat cough, gastric pain, and vomiting tendency [191], however, the extract of *G. glabra* L also has healing properties against gastric ulcer lesion, caused by indomethacin and HCl/Ethanol, in a dose-dependent manner [192]. It has been reported that root of *G. glabra*, also known as licorice or liquorice, derived compounds stimulate the mucus secretion from the stomach, expand the life span of the surface cell of the stomach, and enhance the prostaglandin level which eventually lead to ulcer healing [193]. According to recent studies, the most important bioactive compounds in licorice are flavonoids, triterpenes, and polysaccharides [194]. Glycyrrhizin (molecular formula: C_42_H_62_O_16_) (Figure 1), the major bioactive compound of *G. glabra*, is effective against inflammation and ulcers [195,196]. This triterpenoid saponin-based compound has two isomers such as 18α-GL & 18β-GL and they have anti-fibrogenic properties [197]. Due to the probable antioxidant effect, extracts of licorice had a healing capacity in gastrointestinal ulceration. In the early 1960s, a succinate derivative of glycerrhetinic acid called carbenoxolone (molecular formula: C_34_H_50_O_7_) (Figure 1) was developed in London as an antiulcer drug and was used to assist in the healing of ulcers as the preferred form of licorice [198]. It is determined that *G. glabra* can increase superoxide dismutase effectively as an enzymatic defense system to treat ulcerative colitis induced by acetic acid. Moreover, it played a crucial role in diminishing the levels of NO, TNF-α, and IL-6 in a dose-dependent manner [199]. Anti-inflammatory mechanisms of licorice and *G. glabra* include four major factors: prostaglandin E2 (PGE2), TNF, MMPs, and free radicals (including reactive oxygen, and nitrogen species) were reported based on previous several studies [190].

### 3.21. Hibiscus rosa-sinensis

*H. rosa-sinensis* (family: *Malvaceae*) is locally known as ‘joba’ in Bangladesh and commonly known as ‘China rose’ worldwide [200]. The root, leaf, and flower of *H. rosa-sinensis* contains different active constituents such as tannins, steroids, anthraquinones, essential oils, quinines, mucilage, phenols, reducing sugars, flavonoids, carbohydrates, free amino acids, alkaloids, proteins, terpenoids, cardiac glycosides, and saponins [200]. Traditionally, the root of the plant is used for treating ulcers. The administration of aqueous flower extract from this plant (250 mg/kg) revealed gastric ulcer suppressive activity against pylorus-ligation, aspirin-induced, and ethanol-induced ulcers in vivo and this protective activity occurred due to the presence of flavonoids and tannins as free radical quenchers [201]. Fruits are used externally to cure sprains, wounds, and ulcers [200,202,203].

### 3.22. Centella asiatica

*C. asiatica* of the *Umbelliferae* family is locally known as ‘thankunipata’ in Bangladesh and as ‘Indian pennywort’ worldwide. It originates from the wetlands of Asia [204]. The paste of the leaves of *C. asiatica* is locally used for ulcers and different gastric disorders. Traditionally, this plant has been used to treat diarrhea, rheumatism, skin diseases, syphilis, and inflammation. Several main components i.e., madecassoside, madecassic acid, asiaticoside, and asiatic acid, present in *C. asiatica* were extensively studied for therapeutic purposes including ulcers and inflammatory diseases [205]. Asiaticoside (molecular formula: C_48_H_78_O_19_) (Figure 1), a major active constituent of *C. asiatica*, plays an important role in healing gastric ulcers [204]. Asiaticoside is a glycoside compound that belongs to the triterpenoid group [206]. Acetic acid-induced gastric ulcers in rats were reduced with a lower lesion score, minimized ulcer size, and reduced or absent leucocytes infiltration and submucosal edema, when the plant extract was administered orally. Rats pre-treated with leaf extract exhibited comparatively better protection of the gastric mucosa and had cytoprotective effects [207,208].

### 3.23. Lagenaria siceraria

*L. siceraria* of the *Cucurbitaceae* family is commonly known as ‘bottle gourd’. The fruits, leaves, roots, and seeds of *L. siceraria* contain different active constituents such as vitamin B, amino acids, and ascorbic acid (vitamin C) [157,209]. The leaves are cooked and taken by women as a potherb and to relieve the pain during menstruation. The syrups from the fruits are also used to treat bronchial abnormalities like pectoral cough, asthma, etc. [210,211]. The extract of *L. siceraria* has both strong anti-ulcer and antioxidant activities, though the molecular mechanisms of both anti-ulcer and anti-oxidant activities were not investigated [212]. However, the doses of 250 and 400 mg/kg were safe as there was no indications of signs of toxicity or mortality [212]. From another study, a dose up to 1000 mg/kg could be safe after the repeated administration of methanolic extract of *L. siceraria* fruit for 28 days [213].

### 3.24. Mangifera indica

*M. indica* of the *Anacardiaceae* family is commonly known as the ‘mango’ tree [214]. The fruits, stem barks, heartwoods, leaves, and roots of the plant contain active constituents like triterpenoids, polyphenolics (e.g., mangiferin aglycone), sterols (e.g., mangsterol), alkaloids, saponins, tannins and flavonoids (e.g., mangiferin, mangostin, 29-hydroxy mangiferonic), essential oils (e.g., nerol, elemene, linalool, humulene, ocimene), vitamin A, vitamin C, xanthophylls, and β-carotenes [214]. The leaf extract of *M. indica*, along with rice bran oil, is used traditionally for the treatment of ulcers. The young leaves are also capable of curing dysentery. The seed pulp, along with cornflour, can control diabetes. The extracts of the leaves of the mango plant decreased the ulcer index and showed antiulcer properties to fight against in vivo aspirin-induced gastric ulcer [215,216]. The ripe mango juice is used to tackle heat stroke, which is a fatal life-threatening inflammatory response [217]. The extract from the bark can treat fever, cold, and vomiting [218].

### 3.25. Mimosa pudica

*M. pudica* of the *Fabaceae* family is locally known as ‘lajjaboti’ in Bangladesh and is commonly known as ‘zombie’ or ‘shy plant’ worldwide. The leaf juice or whole plant decoction helps in treating urinary tract infection, dysentery, pain in the body or head, tooth pain, and snakebite injury [218,219]. The fresh leaf and seed decoctions are effectively used in curing intestinal ulcers traditionally [218]. It has been shown that the methanolic extract of *M. pudica* exhibited more gastroprotective properties than chloroform extract after administrating two different doses (100 and 200 mg/kg) for a duration of eight days [220]. It has enhanced gastroprotective properties because of the presence of phyto-constituents (e.g., flavonoids, alkaloids, and tannins) and free radical scavenging activity [220]. The ethanolic extract of *M. pudica*, at the dose of 400 mg/kg, significantly attenuated ulcerative colitis induced by 4% acetic acid and potentially reduced both myeloperoxidase and malondialdehyde in rats when compared to the reference drug prednisolone [221].

### 3.26. Momordica charantia

*M. charantia* is a climbing plant of the *Cucurbitaceae* family. It is often called ‘bitter gourd’ worldwide and as ‘corolla’ in Bangladesh [222,223]. Powder prepared from the whole plant is locally used in treating diversified ulcers [218]. The local people use the unripe fruits as vegetables and cook them. The highest percentage of gastric ulcer inhibition was shown to be 62.85% in the ethanol-induced ulcer model at a dose of 100 mg/kg compared to the standard ranitidine [224]. There was significant healing activity with this plant extract to treat acetic acid-induced gastric ulcers [225]. The extract successfully reduced the ulcer index and inhibited the development of gastric ulcers in all the experimental ulcer models including indomethacin-induced, pylorus-ligated, ethanol-induced, cysteamine-induced duodenal ulcers, and stress-induced ulcer models [226].

### 3.27. Moringa oleifera

*M. oleifera* of the *Moringaceae* family is locally known as ‘shajna’ in Bangladesh and as ‘drum-stick tree’ worldwide [223]. This plant contains active constituents like alkaloids, beta carotene, tannins, beta sitosterol, zeatin, quercetin, flavonoids, kaempferom, protein, vitamins, minerals, amino acids, phenolic acids, natural sugars, phytosterols, saponin, and terpenoids [227,228]. Quercetin (molecular formula: C_15_H_10_O_7_) (Figure 1), a flavonoid compound in *M. oleifera*, showed strong anti-inflammatory activity [229]. The leaf of *M. oleifera* helps in the treatment of ulcers, indigestion, asthma, and sinus pain [219,227,228]. The ethanolic root extract significantly decreased ulcer index, total acidity, and enhanced gastric pH at both doses of 350 and 500 mg/kg. The extract also has anti-secretory and cytoprotective potentiality [230]. The alcoholic leaf extract also resulted in the reduction of acid pepsin secretion and ulcer lesion [38]. The acetone, methanol, chloroform, and petroleum ether extracts of the leaves and fruits of *M. oleifera* were investigated on both gastric and duodenal ulcers using acetic acid, pylorus ligation, indomethacin, ethanol, and cold-restraint stress-induced gastric ulcer and cysteamine-induced duodenal ulcer model [231]. The methanol and acetone extract of the leaves exerted significant gastric antisecretory and gastric cytoprotective effects in pylorus-ligated rats and both ethanol- and indomethacin-induced gastric ulcers, respectively. Compared to the insignificant antiulcer effect of fruit, the leaf extract reduced both cysteamine-induced duodenal ulcers and the stress-induced gastric ulcers remarkably [231].

### 3.28. Psidium guajava

*P. guajava* of the *Myrtaceae* family is commonly known as ‘guava’, which contains different active chemical constituents like tannin, resin, quercetin, crystals of calcium oxalate, guaijaverin, fat, galactose-specific lecithins, cellulose, volatile oil, mineral salts, and flavonoids [228,232]. The decoction prepared from the leaves of *P. guajava* is useful in treating ulcers. The extract of *P. guajava* leaves is also effective in the problems associated with diabetes and stomachache [218,228,233].

### 3.29. Scindapsus officinalis

*S. officinalis* (family: *Araceae*), which is locally known as ‘guruchi lota’, is extensively used by folk medicinal herbalists in Bangladesh in treating ulcers, indigestion, anti-emetic, and helminthiasis [234]. When an in vivo study was conducted to figure out the efficiency of the fruit extract of *S. officinalis* to treat pyloric-ligation-induced gastric ulcers in Wistar rats, the high dose of the extract (500 mg/kg) showed the potential to minimize the ulcer index. The anti-ulcer activity might be ascribed to the free radical scavenging activity [235].

### 3.30. Shorea robusta

*S. robusta* of the *Dipterocarpaceae* family and is commonly known as ‘sal tree’ and locally known as ‘shaal’, in Bangladesh [236]. This plant contains different active constituents like mangiferonic acid, uvaol, ursolic acid, αand β amyrin, asiatic acid, tri, and tetrehydroxyursenoic acid [236]. Ointments prepared from *S. robusta* are traditionally used in curing different ailments such as ulcers, wounds, hemorrhoids, burns, dermatitis, pain, swelling, ear problems, and eye problems. The resin or oleoresin (gum) of the plant is used in curing diarrhea, gonorrhea, and dysentery [236]. Rats received *S. robusta* resin at doses of 150 and 300 mg/kg for treating ethanol and pyloric ligation-induced gastric ulcer, prevented gastric mucosal damage, decreased gastric juice volume, total acidity, and pepsin secretion [237].

### 3.31. Solanum nigrum

*S. nigrum* of the *Solanaceae* family is commonly known as ‘black nightshade’ and locally known as ‘kakmachi’ [209]. The active constituents in this plant are phytosterols, glycoproteins, glycoalkaloids, saponins, polysaccharides, flavonoids, gallic acid, alkaloids, catechin, naringenin, protocatechuic acid, rutin, caffeic acid, and epicatechin [130,202,238,239]. People consume the fresh leaves of *S. nigrum* traditionally in the treatment of intestinal ulcers including hepatotoxicity, body pain, cancer, abdominal problems, and in improving the functions of CNS and the brain [130,202,238].

### 3.32. Syzygium aromaticum

*S. aromaticum* of the *Myrtaceae* family is commonly known as ‘clove’ and locally known as ‘lobongo’ in Bangladesh [240]. *S. aromaticum* contains active volatile and non-volatile constituents like flavonoids, essential oils (e.g., phenyl propanoids, carvacrol, eugenol, thymol, cinnamaldehyde), triterpenes, hydroxycinnamic acid, sterol (e.g., campesterol, stigmasterol) hydroxyphenylpropene, tannins, and hydroxybenzoic acids [130,241,242,243]. The flower bud of the plant helps to prevent ulcers. Traditionally, it is used in dental care for tooth aches, oral ulcers, as a disinfectant in wounds and insect bites, for purifying blood, for maintaining cardiovascular health, as a platelet inhibitor, and in boosting the immune system [236,241].

### 3.33. Terminalia chebula

*T. chebula*, of the *Combretaceae* family, is commonly known as ‘chebulic myrobalan’. In Bangladesh and India, it is well known as ‘haritoki’ [202]. This plant contains diverse bioactive chemical compounds such as chebulic acid, sorbitol, chebulinic acid, tannic acid, chebulagic acid, lucilage, gallic acid, tannin, corilagin, fixed oils, ellagic acid, resin, flavonoids, fructose, amino acids, and sterols [130,202,244]. *T. chebula* mixed with triphala and sindhu salt, is used in treating ulcers and ulcerated wounds. The fruit helps in the treatment of blood dysentery and stomachache. The maceration of the bark of *T. chebula*, in addition to other medicinal plants, helps in waist pain and pain in bones [202,244,245]. Chebulinic acid (molecular formula: C_41_H_32_O_27_), is also called 1,3,6-tri-*O*-galloyl-2,4-chebuloyl-β-d-glucopyranoside, is an ellagitannin abundant in the fruits of *T. chebula* had strong therapeutic efficacy on gastric ulcers [246].

### 3.34. Tinospora cordifolia

*T. cordifolia* (family: *Menispermaceae*), locally known as ‘pipolti’, is traditionally used as a treatment for gastric trouble and ulcers [247]. Moreover, the anti-ulcer efficiency of *T. cordifolia* extracts was assessed in ethanol and the pylorus ligation-induced ulcer model whereas a remarkable reduction of ulcer index, gastric volume, and total acidity supported the efficacy of the extract as an anti-ulcer agent [248]. The major phytocomponents which were isolated from the *T. cordifolia*, sesquiterpene tinocordifolin, tinocordifolioside, sesquiterpene glycoside, arabinogalactan, tinocordiside, makisterone, magnoflorine, and palmatine could play a vital role in reducing the illness [249].

### 3.35. Zingiber officinale

*Z. officinale* of the *Zingiberaceae* family is commonly known as ‘ginger’, which is traditionally used as a medicinal preparation in the treatment of peptic ulcers, diarrhea, allergy, smallpox, asthma, urticaria, fever, impotence, and bronchitis [216,250,251]. Several previous studies in animal models showed that gastrointestinal ulcers were induced by hypothermic restraint stress and NSAIDs [252,253,254]. Zingerone (molecular formula: C_11_H_14_O_3_) (Figure 1), a phenolic active constituent of ginger (9.25%) [255], showed the anti-ulcer efficacy in vivo when Wistar rats were fed with zingeron doses (50, 100, 200 mg/kg), by lowering the number and length of ethanol-induced ulcers in zingerone uptaken groups. The effect of zingeron is protective on the artificially induced ulcer because of its free radical quenching potentiality [256]. Research suggested that ginger constituents ameliorate low-dose aspirin-induced gastric ulceration in the gastrointestinal tract. Therefore, a novel prodrug of aspirin designed as 6-gingerol aspirinate reduced acute stomach injury induced by aspirin in mice [257]. It is proposed that the anti-ulcer and healing capacities of zinger, are achieved due to its strong thromboxane synthetase feature, inhibition of K^+^-ATPase, gastric H^+^, and *H. pylori* growth by phenolic antioxidants [252].

**Figure 2:**
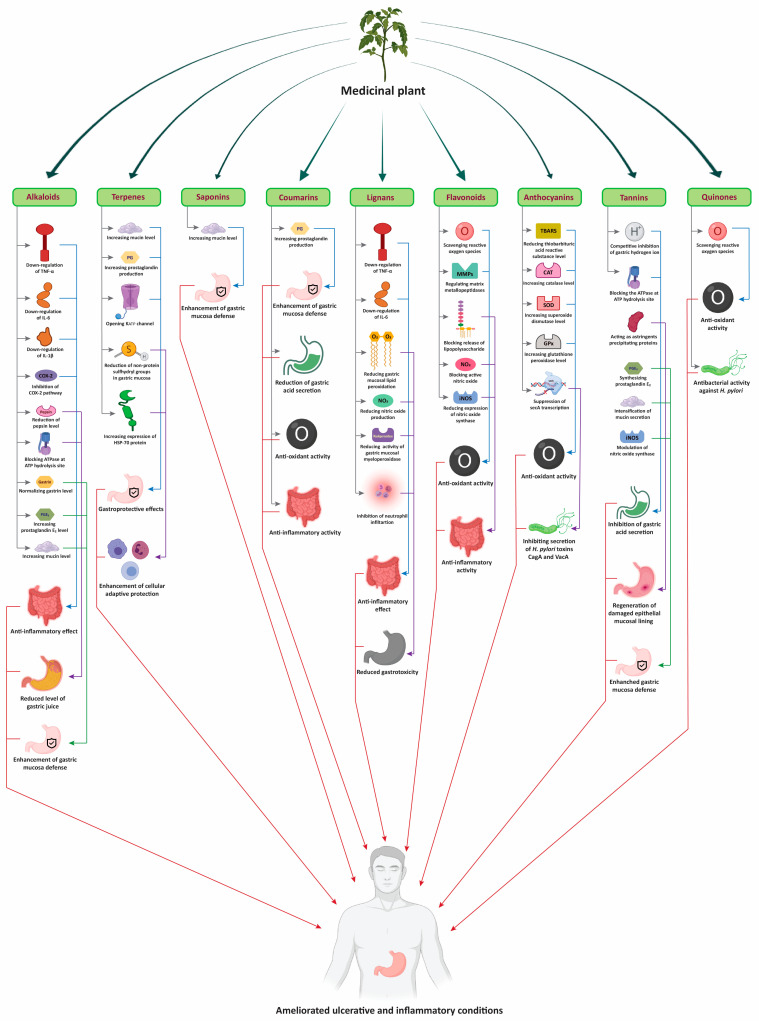
Modes of action of bioactive compounds of medicinal plants to treat ulcers and inflammatory diseases.

All the medicinal plants discussed above show anti-ulcer activity and their uses are in practice among people since ancient time. These plants not only cure ulcers but also show potency against other diseases. The modes of action of bioactive compounds isolated from medicinal plants is summarized in Figure 2. Moreover, other similar medicinal plants with antiulcer and anti-inflammatory activity are summarized in Table 1, with their therapeutic uses and medicinal parts to be used, for easy identification.

## 4. Conclusions and Future Directions

There is a belief that the solutions to all health problems can be found in nature. Currently, humans are victimized by various life-threatening diseases, and in the form of medicinal plant, nature has proven its potency in preventing and curing these diseases. Between 70% and 80% of people throughout the world are solely dependent on primitive or herbal medicines [286]. The worldwide demand for herbal medicine is great and is growing. However, the trials and experiments in this sector are very limited until recently, and this reflects our lack of knowledge about nature. All around the world, there are huge numbers of medicinal plants but the medicinal value of less than one-third of them have been discovered and identified. Still now, more comprehensive, and reliable scientific studies are needed to evaluate and ensure the safety and efficiency of medicinal plants, as well as their potential metabolites in ulcers and inflammatory disease treatment. The quality of the targeted active compounds should be tested repeatedly or batch-to-batch for ensuring reproducibility as well as uniformity of the active components. The implementation of randomized clinical trials overcoming exiting challenges like study design, selection of controls, and active ingredients standardization is urgently needed. International and local regulatory bodies should come forward to facilitate and monitor the clinical trials for achieving a breakthrough outcome.

## Figures and Tables

**Table 1:** Common medicinal plants of Bangladesh used in the treatment of ulcer, inflammatory, and other related diseases.

Name (Family)	Local Name in Bangladesh	Commonly Used Medicinal Parts	Therapeutic Uses	References
*Acacia senegal* (*Leguminosae*)	Babla	Roots, bark, wood leaves, flowers, gums, and seeds	Used to treat ulcers, inflammation, to cure stomach and throat pain	[228,258]
*Acacia farnesiana* (*Leguminosae*)	Belatibabul	Leaves, bark, and flowers	Anti-inflammatory, anti-hepatotoxic, anti-pyritic and anti-ulcerogenic	[259,260]
*Achyranthes aspera* (*Amaranthaseae*)	Apang	Whole plant, roots, and seeds	Used to treat ulcers and inflammation	[261,262]
*Albizia procera* (*Mimosaceae*)	Silkorai	Bark and leaves	Used to treat ulcers, threadworms, skin scabies, and tooth pain	[233]
*Allophylus serratus* (*Sapindaceae*)	Tippani	Leaves, roots, flowers and seeds	Used to treat ulcers, inflammation, and gastrointestinal disorders	[130,263]
*Alstonia scholaris* (*Apocynaceae*)	Chatim	Bark and latex	Used to treat ulcer, dysentery, and rheumatism	[36,264]
*Amaranthus viridis* (*Amaranthaceae*)	Noteyshak	Leaves and seeds	Used to treat ulcer and inflammation	[265]
*Amberboa moschata* (*Asteraceae*)	Jam	Roots	Used to treat ulcers, malignancy, and menstrual disorders	[66]
*Angelica sinensis* (*Apiaceae*)	Not known	Aerial parts	Used to treat ulcerative colitis	[266]
*Anogeissus latifolia* (*Combretacea*)	Dhai	Roots, bark, leaves and fruits	Used to treat inflammation, ulcer, dysentery, hemorrhoids, and liver diseases	[261,267]
*Azadirachta indica* (*Meliaceae*)	Neem	Leaves, roots, seeds, and bark	Anti-inflammatory, anti-pyretic, anti-ulcer, anti-arrhythmic, anti-protozoal, and gastrointestinal disease	[268,269,270]
*Basella alba Linn* (*Basellaceae*)	Puishak	Leaves	Used to treat ulcers and constipation	[259]
*Butea frondosa* (*Leguminosae*)	Palash	Flower, roots, gums, stem, bark and seeds	Anti-ulcerogenic, anti-hemorrhagic activity, and septic sore throats	[36]
*Carissa congesta* (*Apocynaceae*)	Karamcha	Roots and leaves	Used to treat diabetes and ulcers	[157]
*Cissus quadrangularis* (*Vitaceae*)	Harjora or Harbhanga	Stems and rhizomes	Used to treat stomach trouble and ulcers	[135]
*Colocasia esculenta* (*Araceae*)	Mukhikachu	Whole plant	Used to treat tumor, ulcers, cancer, constipation, and indigestion	[157,219,233,271]
*Commiphora mukul* (*Burseraceae*)	Guggul	Guggul gum	Used to treat ulcers, atherosclerosis, rheumatism, and hypercholesterolemia	[228,272]
*Desmostachya bipinnata* (*Gramineae*)	Kush or Durva	Roots	Used to treat ulcers, cancers, and diarrheal disease	[130,273]
*Dyospyros perigrina* (*Ebenaceae*)	Gaab	Fruits and seeds	Used to treat ulcer, diarrhea, dysentery, and wounds	[233]
*Dyospyros philippensis* (*Ebenaceae*)	Boniok	Fruits and seeds	Used to treat ulcer, diarrhea, dysentery, and wounds	[233]
*Euphorbia hirta* (*Euphorbiaceae*)	Bara or Dudhia	Whole plant	Used to treat ulcers, inflammation, and bronchitis	[219,233,273]
*Euphorbia neriifolia* (*Eurphorbiaceae*)	Mansaij or Patasij	Leaves, flowers, fruits, and seeds	Used to treat ulcers, scabies, and schizophrenia	[228,274]
*Excoecaria agallocha* (*Euphorbiaceae*)	Gewa	Leaves, bark, roots	Used to treat microbial infections, cancers, wound and ulcers.	[130,275]
*Ficus religiosa* (*Urticaceae*)	Aswatha or Panbot	Leaves, stem bark, seeds, and roots	Used to treat ulcers, asthma, epilepsy, and inflammatory gastric problems	[276,277]
*Ginkgo biloba* (*Ginkgoaceae*)	Adel or Ginkgo	Root	Applied to cure colitis	[278]
*Heliotropium indicum* (*Boraginaceae*)	Hatisur	Whole plant	Used to treat ulcers, sores, and rheumatism	[233,244]
*Lannea coromandelica* (*Anacardiaceae*)	Jiga	Bark	Anti-eruptions, anti-leprous, anti-ulcer, and ulcerative dyspepsia	[158,233]
*Lawsonia inermis* (*Lythraceae*)	Mehedi	Leaves	Cures wounds and ulcers	[228,244]
*Lens esculenta* (*Leguminosae*)	Masur	Seeds	Used to treat ulcers	[233]
*Lippia nodiflora* (*Verbenaceae*)	Bhui Okar	Leaves and whole plant	Used to treat bronchial problem and ulcers	[259]
*Ludwigia adscendens* (*Onagraceae*)	Keshardam	Whole plant	Used to treat ulcers	[233]
*Mikania micrantha* (*Asteraceae*)	Toopainna Lata	Leaves	Used to treat gastric ulcers and discomfort of digestive tract	[66]
*Nerium indicum* (*Apocynaceae*)	Karabi	Roots, leaves, and whole plant	Anti-ulcer, diuretic, and reduce swellings	[279]
*Oxystelma esculentum* (*Asclepiadaceae*)	Dudhi or Dudhia Lata	Leaves, petiole, stem, roots, and rhizomes	Anti-ulcer, diuretic, and anti-bronchitis activity	[261,280]
*Piper betel* (*Piperaceae*)	Paan	Leaves	Used as digestive aid, anti-oxidant, anti-inflammatory, and analgesic property	[130,281]
*Plumeria alba* (*Apocynaceae*)	Kathgolap	Bark, leaves, flower buds, and latex	Used to treat syphilitic ulcers	[279]
*Polyalthia longifolia* (*Annonaceae*)	Debdaru	Whole plant	Anti-ulcerogenic, hepatoprotective, anti-inflammatory, blood pressure, fever, and moisturizing activity	[282,283]
*Scoparia dulcis* (*Scrophulariaceae*)	Michri Dana	Leaves	Used in the treatment of inflammation and nerve problems	[244]
*Sesbania grandiflora* (*Leguminosae*)	Bokful	Leaves	Used to treat ulcers	[284,285]
*Smilax ovalifolia* (* Smilacaceae*)	Kumarilata	Leaves and stems	Used to treat ulcers	[66]
*Vernonia patula* (*Asteraceae*)	Kuksim	Aerial parts	Anti-ulcer, anti-inflammatory, and anti-dropsy	[219]
*Xanthium indicum* (*Asteraceae*)	Ghagra	Stems, roots, fruits, leaves, and whole plant	Used to treat ulcers, tumors, and smallpox	[219]
*Zizyphus mauritiana* (*Rhamnaceae*)	Ber or Kool	Roots and bark	Used to treat wounds, fever, ulcers, and diarrhea	[233]

## Data Availability

Not applicable.

## References

[1] Bishayee A., Sethi G. (2016). Bioactive natural products in cancer prevention and therapy: Progress and promise. Semin. Cancer Biol..

[2] Mintah S.O., Asafo-Agyei T., Archer M.-A., Junior P.A.-A., Boamah D., Kumadoh D., Appiah A., Ocloo A., Boakye Y.D., Agyare C. (2019). Medicinal plants for treatment of prevalent diseases. Pharmacognosy-Medicinal Plants.

[3] Sumner J. (2000). The Natural History of Medicinal Plants.

[4] Arif T., Bhosale J.D., Kumar N., Mandal T.K., Bendre R.S., Lavekar G.S., Dabur R. (2009). Natural products—Antifungal agents derived from plants. J. Asian Nat. Prod. Res..

[5] Cragg G.M., Newman D.J. (2013). Natural products: A continuing source of novel drug leads. Biochim. Biophys. Acta.

[6] Imadi S.R., Mahmood I., Gul A., Ozturk M., Hakeem K.R. (2018). Medicinal Plants Against Cancer. Plant and Human Health, Volume 1: Ethnobotany and Physiology.

[7] Laila U., Akram M., Shariati M.A., Hashmi A.M., Akhtar N., Tahir I.M., Ghauri A.O., Munir N., Riaz M., Akhter N. (2019). Role of medicinal plants in HIV/AIDS therapy. Clin. Exp. Pharm. Physiol..

[8] Siddiqui M.H., Alamri S.A., Al-Whaibi M.H., Hussain Z., Ali H.M., El-Zaidy M.E. (2017). A mini-review of anti-hepatitis B virus activity of medicinal plants. Biotechnol. Biotechnol. Equip..

[9] Dias D.A., Urban S., Roessner U. (2012). A historical overview of natural products in drug discovery. Metabolites.

[10] Guaadaoui A., Benaicha S., Elmajdoub N., Bellaoui M., Hamal A. (2014). What is a bioactive compound? A combined definition for a preliminary consensus. Int. J. Nutr. Food Sci..

[11] Mukherjee P.K. (2002). Quality Control of Herbal Drugs: An Approach to Evaluation of Botanicals.

[12] Jamshidi-Kia F., Lorigooini Z., Amini-Khoei H. (2018). Medicinal plants: Past history and future perspective. J. Herbmed. Pharm..

[13] Parveen A., Parveen B., Parveen R., Ahmad S. (2015). Challenges and guidelines for clinical trial of herbal drugs. J. Pharm. Bioallied Sci..

[14] Wakdikar S. (2004). Global health care challenge: Indian experiences and new prescriptions. Electron. J. Biotechnol..

[15] Lyubchyk S., Shapovalova O., Lygina O., Oliveira M.C., Appazov N., Lyubchyk A., Charmier A.J., Lyubchik S., Pombeiro A.J. (2019). Integrated Green Chemical Approach to the Medicinal Plant Carpobrotus edulis Processing. Sci. Rep..

[16] Venkatachalapathi A., Sangeeth T., Paulsamy S. (2015). Ethnobotanical informations on the species of selected areas in Nilgiri Biosphere Reserve, the Western Ghats, India. J. Res. Biol..

[17] Curtis P., Gaylord S. (2005). Safety Issues in the Interaction of Conventional, Complementary, and Alternative Health Care. Complement. Health Pr. Rev..

[18] Kalra P., Sharma S., Kumar S. (2011). Antiulcer effect of the methanolic extract of *Tamarindus indica* seeds in different experimental models. J. Pharm. Bioallied Sci..

[19] Koo Y.E., Song J., Bae S. (2018). Use of plant and herb derived medicine for therapeutic usage in cardiology. Medicines.

[20] Triantafyllidi A., Xanthos T., Papalois A., Triantafillidis J.K. (2015). Herbal and plant therapy in patients with inflammatory bowel disease. Ann. Gastroenterol..

[21] Santana M.T., Cercato L.M., Oliveira J.P., Camargo E.A. (2017). Medicinal plants in the treatment of colitis: Evidence from preclinical studies. Planta Med..

[22] Wan P., Chen H., Guo Y., Bai A.-P. (2014). Advances in treatment of ulcerative colitis with herbs: From bench to bedside. World J. Gastroenterol..

[23] Oguntibeju O.O. (2018). Medicinal plants with anti-inflammatory activities from selected countries and regions of Africa. J. Inflamm. Res..

[24] Laveti D., Kumar M., Hemalatha R., Sistla R., Gm Naidu V., Talla V., Verma V., Kaur N., Nagpal R. (2013). Anti-inflammatory treatments for chronic diseases: A review. Inflamm. Allergy Drug Targets.

[25] Sun Q., Zhu J., Cao F., Chen F. (2017). Anti-inflammatory properties of extracts from Chimonanthus nitens Oliv. leaf. PLoS ONE.

[26] Schett G., Neurath M.F. (2018). Resolution of chronic inflammatory disease: Universal and tissue-specific concepts. Nat. Commun..

[27] Moura F.A., Goulart M.O.F. (2017). Inflammatory Bowel Diseases: The Crosslink Between Risk Factors and Antioxidant Therapy. Gastrointestinal Tissue.

[28] Awaad A.S., El-Meligy R.M., Soliman G.A. (2013). Natural products in treatment of ulcerative colitis and peptic ulcer. J. Saudi Chem. Soc..

[29] Rau W., Hohaus C., Jessen E. (2019). A differential approach to form and site of peptic ulcer. Sci. Rep..

[30] Lin H.-Y., Weng S.-F., Lin H.-J., Hsu C.-C., Wang J.-J., Su S.-B., Guo H.-R., Huang C.-C. (2015). Peptic ulcer disease in healthcare workers: A nationwide population-based cohort study. PLoS ONE.

[31] Narayanan M., Reddy K.M., Marsicano E. (2018). Peptic ulcer disease and Helicobacter pylori infection. Mol. Med..

[32] Drini M. (2017). Peptic ulcer disease and non-steroidal anti-inflammatory drugs. Aust. Prescr..

[33] Sung J., Kuipers E., El-Serag H. (2009). Systematic review: The global incidence and prevalence of peptic ulcer disease. Aliment. Pharm. Ther..

[34] Lehours P., Ferrero R.L. (2019). Helicobacter: Inflammation, immunology, and vaccines. Helicobacter.

[35] Priyanka V. (2015). Some of the medicinal plants with anti-ulcer activity—A review. J. Pharm. Sci. Res..

[36] Gadekar R., Singour P., Chaurasiya P., Pawar R., Patil U. (2010). A potential of some medicinal plants as an antiulcer agents. Pharm. Rev..

[37] Goel R., Sairam K. (2002). Anti-ulcer drugs from indigenous sources with emphasis on Musa sapientum, tamrahbasma, *Asparagus racemosus* and *Zingiber officinale*. Indian J. Pharm..

[38] Verma V.K., Singh N., Saxena P., Singh R. (2012). Anti-ulcer and antioxidant activity of Moringa oleifera (Lam) leaves against aspirin and ethanol induced gastric ulcer in rats. Int. Res. J. Pharm..

[39] Sharifi-Rad M., Fokou P.V.T., Sharopov F., Martorell M., Ademiluyi A.O., Rajkovic J., Salehi B., Martins N., Iriti M., Sharifi-Rad J. (2018). Antiulcer agents: From plant extracts to phytochemicals in healing promotion. Molecules.

[40] Fürst R., Zündorf I. (2014). Plant-derived anti-inflammatory compounds: Hopes and disappointments regarding the translation of preclinical knowledge into clinical progress. Mediat. Inflamm..

[41] Jantan I., Ahmad W., Bukhari S.N. (2015). Plant-derived immunomodulators: An insight on their preclinical evaluation and clinical trials. Front. Plant Sci..

[42] Triantafillidis J., Stanciu C. (2012). Inflammatory Bowel Disease: Etiopathogenesis, Diagnosis, Treatment.

[43] Harding S.D., Sharman J.L., Faccenda E., Southan C., Pawson A.J., Ireland S., Gray A.J.G., Bruce L., Alexander S.P.H., Anderton S. (2018). The iuphar/bps guide to pharmacology in 2018: Updates and expansion to encompass the new guide to immunopharmacology. Nucleic Acids Res..

[44] Yang M., He M., Zhao M., Zou B., Liu J., Luo L.-M., Li Q.-L., He J.-H., Lei P.-G. (2017). Proton pump inhibitors for preventing non-steroidal anti-inflammatory drug induced gastrointestinal toxicity: A systematic review. Curr. Med. Res. Opin..

[45] Ghlichloo I., Gerriets V. (2021). Nonsteroidal Anti-inflammatory Drugs (NSAIDs). StatPearls.

[46] Manandhar B., Paudel K.R., Sharma B., Karki R. (2018). Phytochemical profile and pharmacological activity of Aegle marmelos Linn. J. Integr. Med..

[47] Chakraborty P., Banerjee S., Kumar S., Sadhukhan S., Halder G. (2018). Elucidation of ibuprofen uptake capability of raw and steam activated biochar of Aegle marmelos shell: Isotherm, kinetics, thermodynamics and cost estimation. Process. Saf. Environ. Prot..

[48] Jatav S., Pandey N., Dwivedi P., Bansal R., Ahluwalia V., Tiwari V.K., Mishra B.B. (2019). Isolation of a new flavonoid and waste to wealth recovery of 6-O-Ascorbyl Esters from Seeds of Aegle marmelos (family-Rutaceae). Nat. Prod. Res..

[49] Patil M., Patil R., Maheshwari V. (2015). Biological activities and identification of bioactive metabolite from endophytic Aspergillus flavus L7 isolated from Aegle marmelos. Curr. Microbiol..

[50] Daniel M. (2006). Medicinal Plants: Chemistry and Properties.

[51] Kurian J. (2012). Ethno-medicinal plants of India, Thailand and Vietnam. J. Biodivers..

[52] Bramhachari P., Reddy Y., Kotresha D., Varaprasad B. (2010). Phytochemical examination, antioxidant and radical scavenging activity *of Aegle marmelos* (L.) Correa extracts. J. Pharm. Res..

[53] Verma R.S., Padalia R.C., Chauhan A. (2014). Essential oil composition of *Aegle marmelos* (L.) Correa: Chemotypic and seasonal variations. J. Sci. Food Agric..

[54] Chakthong S., Weaaryee P., Puangphet P., Mahabusarakam W., Plodpai P., Voravuthikunchai S.P., Kanjana-Opas A. (2012). Alkaloid and coumarins from the green fruits of Aegle marmelos. Phytochemistry.

[55] Samarasekera J.R., Khambay B.P., Hemalal K.P. (2004). A new insecticidal protolimonoid from *Aegle marmelos*. Nat. Prod. Res..

[56] Singh R. (2019). Ethno-medicinal and Pharmacological activities of *Aegle marmelos* (Linn.) Corr: A review. Pharma Innov. J..

[57] Rahman S., Quader M.R., Khan M.I. (2018). Prevention of peptic ulcer by aqueous extract of Aegle marmelos leaf in rats. Imc J. Med. Sci..

[58] Sharmin Rahman D., Quader M.R., Sharmin R., Momtaz A., Sharmin K., Eva E.O., Mosaddek A.S.M. (2016). Evaluation of Anti Ulcer Activity of Ethanolic Extract of Aegle Marmelos Leaves on Rats. ARC J. Dent. Sci..

[59] Ramakrishna Y.G., Savithri K., Kist M., Devaraj S.N. (2015). Aegle marmelos fruit extract attenuates Helicobacter pylori Lipopolysaccharide induced oxidative stress in Sprague Dawley rats. BMC Complement. Altern. Med..

[60] Dhuley J. (2003). Investigation on the gastroprotective and antidiarrhoeal properties of Aegle marmelos unripe fruit extract. Hindustan Antibiot. Bull..

[61] Rahman S., Parvin R. (2014). Therapeutic potential of *Aegle marmelos* (L.)-An overview. Asian Pac. J. Trop. Dis..

[62] Pathirana C.K., Madhujith T., Eeswara J. (2020). Bael (*Aegle marmelos* L. Corrêa), a Medicinal Tree with Immense Economic Potentials. Adv. Agric..

[63] Pynam H., Dharmesh S.M. (2018). Antioxidant and anti-inflammatory properties of marmelosin from Bael (*Aegle marmelos* L.); Inhibition of TNF-α mediated inflammatory/tumor markers. Biomed. Pharm..

[64] Longo B., Sommerfeld E.P., Somensi L.B., Mariano L.N.B., Boeing T., de Andrade S.F., de Souza P., da Silva L.M. (2021). Dual role of eugenol on chronic gastric ulcer in rats: Low-dose healing efficacy and the worsening gastric lesion in high doses. Chemi Biol. Interact..

[65] Chniguir A., Pintard C., Liu D., Dang P.M.-C., El-Benna J., Bachoual R. (2019). Eugenol prevents fMLF-induced superoxide anion production in human neutrophils by inhibiting ERK1/2 signaling pathway and p47phox phosphorylation. Sci. Rep..

[66] Faruque M.O., Feng G., Khan M.N.A., Barlow J.W., Ankhi U.R., Hu S., Kamaruzzaman M., Uddin S.B., Hu X. (2019). Qualitative and quantitative ethnobotanical study of the Pangkhua community in Bilaichari Upazilla, Rangamati District, Bangladesh. J. Ethnobiol. Ethnomed..

[67] Maan A.A., Nazir A., Khan M.K.I., Ahmad T., Zia R., Murid M., Abrar M. (2018). The therapeutic properties and applications of aloe vera: A review. J. Herb. Med..

[68] Chowdhury M.S.H., Koike M., Muhammed N., Halim M.A., Saha N., Kobayashi H. (2009). Use of plants in healthcare: A traditional ethno-medicinal practice in rural areas of southeastern Bangladesh. Int. J. Biodivers. Sci. Manag..

[69] Suvitayavat W., Sumrongkit C., Thirawarapan S., Bunyapraphatsara N. (2004). Effects of Aloe preparation on the histamine-induced gastric secretion in rats. J. Ethnopharmacol..

[70] Wani M.Y., Hasan N., Malik M.A. (2010). Chitosan and Aloe vera: Two gifts of nature. J. Dispers. Sci. Tech..

[71] El-Batal A.I., Ahmed S.F. (2018). Therapeutic effect of Aloe vera and silver nanoparticles on acid-induced oral ulcer in gamma-irradiated mice. Braz. Oral Res..

[72] Borra S.K., Lagisetty R.K., Mallela G.R. (2011). Anti-ulcer effect of Aloe vera in non-steroidal anti-inflammatory drug induced peptic ulcers in rats. Afr. J. Pharm. Pharm..

[73] Koo M. (1994). Aloe vera: Antiulcer and antidiabetic effects. Phytother. Res..

[74] Bahrami G., Malekshahi H., Miraghaee S., Madani H., Babaei A., Mohammadi B., Hatami R. (2020). Protective and Therapeutic Effects of Aloe Vera Gel on Ulcerative Colitis Induced by Acetic Acid in Rats. Clin. Nutr. Res..

[75] Foster M., Hunter D., Samman S. (2011). Evaluation of the nutritional and metabolic effects of Aloe vera. Herbal Medicine: Biomolecular and Clinical Aspects.

[76] Gai L., Chu L., Xia R., Chen Q., Sun X. (2019). Barbaloin attenuates mucosal damage in experimental models of rat colitis by regulating inflammation and the AMPK signaling pathway. Med. Sci. Monit. Int. Med. J. Eexperi. Clin. Res..

[77] Rahman M.A., Uddin S., Wilcock C. (2007). Medicinal plants used by Chakma tribe in Hill Tracts districts of Bangladesh. Indian J. Tradit. Knowl..

[78] Ghosh S., Rangan L. (2013). Alpinia: The gold mine of future therapeutics. 3Biotech.

[79] Fan G.-j., Kang Y.-H., Han Y.N., Han B.H. (2007). Platelet-activating factor (PAF) receptor binding antagonists from Alpinia officinarum. Bioorganic Med. Chem. Lett..

[80] Lee J., Kim K.A., Jeong S., Lee S., Park H.J., Kim N.J., Lim S. (2009). Anti-inflammatory, anti-nociceptive, and anti-psychiatric effects by the rhizomes of Alpinia officinarum on complete Freund’s adjuvant-induced arthritis in rats. J. Ethnopharmacol..

[81] Das B.K., Fatema U.K., Hossain M.S., Rahman R., Fatema K. (2014). Anti-inflammatory and analgesic activities of Alpinia nigra fruit extract in laboratory animals. Eur. J. Med. Plants.

[82] Williams R.J., Spencer J.P., Rice-Evans C. (2004). Flavonoids: Antioxidants or signalling molecules?. Free Radic. Biol. Med..

[83] Khan E.A., Khan T.A. (2018). Adsorption of methyl red on activated carbon derived from custard apple (*Annona squamosa*) fruit shell: Equilibrium isotherm and kinetic studies. J. Mol. Liq..

[84] Luca S.V., Macovei I., Bujor A., Miron A., Skalicka-Woźniak K., Aprotosoaie A.C., Trifan A. (2020). Bioactivity of dietary polyphenols: The role of metabolites. Crit. Rev. Food Sci. Nutr..

[85] Yang Y.L., Chang F.R., Wu Y.C. (2004). Annosqualine: A novel alkaloid from the stems of Annona squamosa. Helv. Chim. Acta.

[86] Panda S., Kar A. (2007). Antidiabetic and antioxidative effects of Annona squamosa leaves are possibly mediated through quercetin-3-O-glucoside. Biofactors.

[87] Zahid M., Arif M., Rahman M.A., Mujahid M. (2018). Hepatoprotective and antioxidant activities of Annona squamosa seed extract against alcohol-induced liver injury in Sprague Dawley rats. Drug Chem. Ttoxicol..

[88] Chavan M., Shinde D., Nirmal S. (2006). Major volatile constituents of *Annona squamosa* L. bark. Nat. Prod. Res..

[89] Yadav D.K., Singh N., Dev K., Sharma R., Sahai M., Palit G., Maurya R. (2011). Anti-ulcer constituents of Annona squamosa twigs. Fitoterapia.

[90] Singh D.P., Mishra B., Mishra R. (2012). Anti-nociceptive and anti-inflammatory activity of *Annona squamosa* L. leaf extract in mice and rats. Res. J. Pharm. Phytochem..

[91] Panda S., Kar A. (2007). Annona squamosa seed extract in the regulation of hyperthyroidism and lipid-peroxidation in mice: Possible involvement of quercetin. Phytomedicine.

[92] Kooti W., Farokhipour M., Asadzadeh Z., Ashtary-Larky D., Asadi-Samani M. (2016). The role of medicinal plants in the treatment of diabetes: A systematic review. Electron. Physician.

[93] Mondal S., Bhar K., Mahapatra A.S., Mukherjee J., Mondal P., Rahaman S.T., Nair A.P. (2020). “Haripriya” god’s favorite: Anthocephalus cadamba (Roxb.) Miq.-At a glance. Pharm. Res..

[94] Inaparthi V.K., Babu P.N., Prasad K., Nagaraju B., Prasanthi K. (2014). Evaluation of anti-ulcer and in-vitro antioxidant activities of aqueous and methanolic extracts of neolamarckia cadamba leaves and bark in wistar albino rats. Int. J. Pharm. Sci. Res..

[95] Subhan N., Hasan R., Hossain M., Akter R., Majumder M.M., Rahman M.M., Ahmed K., Ghani A., Alam M.A. (2009). Antinociceptive and gastro-protective effect of the ethanolic extract of the flowering top of Anthocephalus Cadamba Roxb. Orient. Pharm. Exp. Med..

[96] Bachhav R., Buchake V., Saudagar R. (2009). Analgesic and anti-inflammatory activities of Anthocephalus cadamba roxb. leaves in wistar rats. Res. J. Pharm. Technol..

[97] Ambujakshi H., Antony S., Kanchana Y., Patel R., Thakkar H. (2009). Analgesic activity of Anthocephalus cadamba leaf extract. J. Pharm. Res..

[98] Sumanta M., Dash G., Suman A. (2009). Analgesic, anti-inflammatory and antipyretic studies of Neolamarckia cadamba barks. J. Pharm. Res..

[99] Chandrashekar K.S., Prasanna K.S., Abinash B. (2010). Anti-inflammatory effect of the methanol extract from Anthocephalus cadamba stem bark in animal models. Int. J. Plant. Biol..

[100] Naz R., Ayub H., Nawaz S., Islam Z.U., Yasmin T., Bano A., Wakeel A., Zia S., Roberts T.H. (2017). Antimicrobial activity, toxicity and anti-inflammatory potential of methanolic extracts of four ethnomedicinal plant species from Punjab, Pakistan. BMC Complement. Altern. Med..

[101] Kuo P.-C., Li Y.-C., Wu T.-S. (2012). Chemical Constituents and Pharmacology of the Aristolochia (馬兜鈴 mădōu ling) species. J. Tradit. Complement. Med..

[102] Desai D.C., Jacob J., Almeida A., Kshirsagar R., Manju S. (2014). Isolation, structural elucidation and anti-inflammatory activity of astragalin,(−) hinokinin, aristolactam I and aristolochic acids (I & II) from Aristolochia indica. Nat. Prod. Res..

[103] Mathew J.E., Kaitheri S.K., DinakaranVachala S., Jose M. (2011). Anti-inflammatory, antipruritic and mast cell stabilizing activity of Aristolochia indica. Iran. J. Basic Med. Sci..

[104] Rahmatullah M., Rahman A., Uddin F., Hasan M., Khatun A., Bashar A.A., Ahsan S., Mou S.M., Begum R., Jahan R. (2011). An ethnomedicinal survey conducted amongst folk medicinal practitioners in the two southern districts of Noakhali and Feni, Bangladesh. Adv. Nat. Appl. Sci..

[105] Prakash O., Kumar R., Chandra D., Kumar A., Kumar P. (2015). Effect of Artocarpus heterophyllus Lam.(Jackfruit) on Indomethacin-Induced ulcer model in albino rats. Der. Pharm. Lett..

[106] Yao X., Wu D., Dong N., Ouyang P., Pu J., Hu Q., Wang J., Lu W., Huang J. (2016). Moracin C, a phenolic compound isolated from Artocarpus heterophyllus, suppresses lipopolysaccharide-activated inflammatory responses in murine raw264. 7 macrophages. Int. J. Mol. Sci..

[107] Jashni H.K., Jahromi H.K., Ranjbary A.G., Jahromi Z.K., Kherameh Z.K. (2016). Effects of aqueous extract from *Asparagus officinalis* L. roots on hypothalamic-pituitary-gonadal axis hormone levels and the number of ovarian follicles in adult rats. Int. J. Reprod. Biomed..

[108] Singh L. (2018). Asparagus racemosus: The plant with immense medicinal potential. J. Pharm. Phytochem..

[109] Goyal R., Singh J., Lal H. (2003). Asparagus racemosus-an update. Indian J. Med. Sci..

[110] Joshi R.K. (2016). Asparagus racemosus (Shatawari), phytoconstituents and medicinal importance, future source of economy by cultivation in Uttrakhand: A review. Int. J. Herb. Med..

[111] Thakur S., Sharma D. (2015). Review on medicinal plant: Asparagus adscendens Roxb. Int. J. Pharma. Sci. Health Care.

[112] Sairam K., Priyambada S., Aryya N., Goel R. (2003). Gastroduodenal ulcer protective activity of Asparagus racemosus: An experimental, biochemical and histological study. J. Ethnopharmacol..

[113] Bhatnagar M., Sisodia S. (2006). Antisecretory and antiulcer activity of Asparagus racemosus Willd. against indomethacin plus pyloric ligation-induced gastric ulcer in rats. J. Herb. Ppharmacother..

[114] Mashele S.S. (2019). Medicinal Properties of Selected Asparagus Species: A Review. Phytochemicals in Human Health.

[115] Kujala T.S., Vienola M.S., Klika K.D., Loponen J.M., Pihlaja K. (2002). Betalain and phenolic compositions of four beetroot (*Beta vulgaris*) cultivars. Eur. Food Res. Technol..

[116] Samyuktha K., Chinnala K.M., Prathiba G., Rajendhar D., Reddy P.S. (2017). Evaluation of anti ulcer activity of ethanolic root extract of Beta vulgaris in rats. Int. J. Basic Clin. Pharm..

[117] Hijazi H.H., Rezq A.A., Elgazar A.F. (2018). Studying Anti-inflammatory and Anti-Peptic Ulcer Effects of Aqueous Extract of Red Beetroots on Male Rats. Int. J. Pharm. Life Sci..

[118] Neha P., Sk J., Nk J., Hk J. (2018). Chemical and functional properties of Beetroot (*Beta vulgaris* L.) for product development: A review. Int. J. Chem. Stud..

[119] El-Beltagi H.S., Mohamed H.I., Megahed B.M., Gamal M., Safwat G. (2018). Evaluation of some chemical constituents, antioxidant, antibacterial and anticancer activities of *Beta vulgaris* L. root. Fresenius Environ. Bull..

[120] Ceclu L., Nistor O. (2020). Red Beetroot: Composition and Health Effects—A Review. J. Nutr. Med. Diet. Care.

[121] Nadipelly J., Kothapalli J. (2016). Antiulcer activity of petroleum ether extract of *Beta vulgaris* (L.). Int. J. Res.Dev. Pharm. Life Sci..

[122] Jain S., Garg V.K., Sharma P.K. (2011). Anti-inflammatory activity of aqueous extract of *Beta vulgaris* L.. J. Basic Clin. Pharm..

[123] Chaudhary P.H., Tawar M.G. (2019). Pharmacognostic and Phytopharmacological Overview on Bombax ceiba. Syst. Rev. Pharm..

[124] Panda D. (2018). Ethnobotanical study of medicinal plants in Jajpur district of Odisha, India. J. Pharm. Phytochem..

[125] Navdeep Ranjan S.K.S.a.C.K. (2018). Role of medicinal plants in traditional medicine system in bihar—A review. World J. Pharm. Res..

[126] Dar A., Faizi S., Naqvi S., Roome T., Zikr-ur-Rehman S., Ali M., Firdous S., Moin S.T. (2005). Analgesic and antioxidant activity of mangiferin and its derivatives: The structure activity relationship. Biol. Pharm. Bull..

[127] Jagtap A., Niphadkar P., Phadke A. (2011). Protective effect of aqueous extract of Bombax malabaricum DC on experimental models of inflammatory bowel disease in rats and mice. Indian J. Exp. Biol..

[128] Hussain L., Akash M., Naseem S., Rehman K., Ahmed K.Z. (2015). Anti-ulcerogenic effects of Salmalia malabarica in gastric ulceration-pilot study. Adv. Clin. Exp. Med..

[129] Barakat M., El-Boghdady N., Farrag E., Said A., Shaker S. (2019). Protective and curative effects of Bombax ceiba flower and Ziziphus spina christi fruit extracts on gastric ulcer. J. Biol. Sci..

[130] Srinivas T.L., Lakshmi S.M., Shama S.N., Reddy G.K., Prasanna K. (2013). Medicinal plants as anti-ulcer agents. J. Pharm. Phytochem..

[131] Jaikumar K., Sheik Noor Mohamed M., John Wyson W., Deventhiran M., Anand D., Saravanan P. (2017). Phytochemical analysis of *Caesalpinia pulcherrima* (L.) *Sw.* Leaf extract using GC-MS analysis. Int. J. Pharm. Sci. Drug Res..

[132] Gilani S.M.U., Ahmed S., Baig S.G., Hasan M.M. (2019). Ethnopharmacognosy, phytochemistry and pharmacology of genus Caesalpinia: A review. J. Pharm. Phytochem..

[133] Marques F.d.C.J., da Silva Pantoja P., Matos V.E.A., Silva R.O., Damasceno S.R.B., Franco Á.X., Alves R.C., Justino P.F.C., de Souza M.H.L.P., Feitosa J.P.A. (2019). Galactomannan from the seeds of Caesalpinia pulcherrima prevents indomethacin-induced gastrointestinal damage via neutrophil migration. Int. J. Biol. Macromol..

[134] Sharma V., Rajani G. (2011). Evaluation of Caesalpinia pulcherrima Linn. for anti-inflammatory and antiulcer activities. Indian J. Pharm..

[135] Khan M.A., Islam M.K., Siraj M.A., Saha S., Barman A.K., Awang K., Rahman M.M., Shilpi J.A., Jahan R., Islam E. (2015). Ethnomedicinal survey of various communities residing in Garo Hills of Durgapur, Bangladesh. J. Ethnobiol. Ethnomed..

[136] Chandra P., Kishore K., Ghosh A.K. (2015). Evaluation of antacid capacity and antiulcer activity of *Calendula officinalis* L. in experimental rats. Orient. Pharm. Exp. Med..

[137] Yadav A.K., Pushpesh K., Jain P., Chandana V., Tiwari S., Singh V. (2016). Investigation of Calendula officinalis whole plant as a gastroprotective and antioxidant in peptic ulcer. Braz. J. Med. Health Res..

[138] Preethi K.C., Kuttan G., Kuttan R. (2009). Anti-inflammatory activity of flower extract of Calendula officinalis Linn. and its possible mechanism of action. Indian J. Exp. Biol..

[139] Mali R.P., Rao P.S., Jadhav R. (2019). A Review on Pharmacological Activities of Calotropis Procera. J. Drug Deliv. Thera..

[140] Shamim S.A., Fatima L. (2019). Pharmacological actions and therapeutic uses of Aak (*Calotropis procera*): A Review. Pharma Innov. J..

[141] Mandaville J.P. (2019). Bedouin Ethnobotany: Plant Concepts and Uses in a Desert Pastoral World.

[142] Meena A.K., Yadav A., Rao M. (2011). Ayurvedic uses and pharmacological activities of *Calotropis procera* Linn. Asian J. Tradit. Med..

[143] Tour N.S., Talele G.S. (2011). Gastric antiulcer and antiinflammatory activities of *Calotropis procera* stem bark. Rev. Bras. Farmacogn..

[144] Al-Taweel A.M., Perveen S., Fawzy G.A., Rehman A.U., Khan A., Mehmood R., Fadda L.M. (2017). Evaluation of antiulcer and cytotoxic potential of the leaf, flower, and fruit extracts of *Calotropis procera* and isolation of a new lignan glycoside. Evid. Based Complement. Altern. Med..

[145] Kumar V.L., Pandey A., Verma S., Das P. (2019). Protection afforded by methanol extract of *Calotropis procera* latex in experimental model of colitis is mediated through inhibition of oxidative stress and pro-inflammatory signaling. Biomed. Pharm..

[146] Naveed M., BiBi J., Kamboh A.A., Suheryani I., Kakar I., Fazlani S.A., FangFang X., Yunjuan L., Kakar M.U., El-Hack M.E.A. (2018). Pharmacological values and therapeutic properties of black tea (*Camellia sinensis*): A comprehensive overview. Biomed. Pharm..

[147] Koch W., Zagórska J., Marzec Z., Kukula-Koch W. (2019). Applications of Tea (*Camellia sinensis*) and Its Active Constituents in Cosmetics. Molecules.

[148] Zhang L., Ho C.T., Zhou J., Santos J.S., Armstrong L., Granato D. (2019). Chemistry and biological activities of processed *Camellia sinensis* teas: A comprehensive review. Compr. Rev. Food Sci. Food Saf..

[149] Aboulwafa M.M., Youssef F.S., Gad H.A., Altyar A.E., Al-Azizi M.M., Ashour M.L. (2019). A comprehensive insight on the health benefits and phytoconstituents of *Camellia sinensis* and recent approaches for its quality control. Antioxidants.

[150] Shivashankara A.R., Rao S., George T., Abraham S., Colin M.D., Palatty P.L., Baliga M.S. (2019). Tea (*Camellia sinensis* L. Kuntze) as Hepatoprotective Agent: A Revisit. Dietary Interventions in Liver Disease.

[151] Scoparo C.T., Souza L.M., Dartora N., Sassaki G.L., Santana-Filho A.P., Werner M.F.P., Borato D.G., Baggio C.H., Iacomini M. (2016). Chemical characterization of heteropolysaccharides from green and black teas (*Camellia sinensis*) and their anti-ulcer effect. Int. J. Biol. Macromol..

[152] Adhikary B., Yadav S.K., Roy K., Bandyopadhyay S.K., Chattopadhyay S. (2011). Black tea and theaflavins assist healing of indomethacin-induced gastric ulceration in mice by antioxidative action. Evid. Based Complement. Altern. Med..

[153] Adhikary B., Yadav S.K., Chand S., Bandyopadhyay S.K., Chattopadhyay S. (2011). Black tea and theaflavins suppress various inflammatory modulators and i-NOS mediated nitric oxide synthesis during gastric ulcer healing. Free Radic. Res..

[154] Bae J., Kim N., Shin Y., Kim S.-Y., Kim Y.-J. (2020). Activity of catechins and their applications. Biomed. Derm..

[155] Navarro-Martínez M.D., Navarro-Perán E., Cabezas-Herrera J., Ruiz-Gómez J., García-Cánovas F., Rodríguez-López J.N. (2005). Antifolate activity of epigallocatechin gallate against Stenotrophomonas maltophilia. Antimicrob. Agents Chemother..

[156] Adcocks C., Collin P., Buttle D.J. (2002). Catechins from green tea (*Camellia sinensis*) inhibit bovine and human cartilage proteoglycan and type II collagen degradation in vitro. J. Nutr..

[157] Hasan M.M., Hossain S.A., Ali M.A., Alamgir A. (2014). Medicinal plant diversity in Chittagong, Bangladesh: A database of 100 medicinal plants. J. Sci. Innov. Res..

[158] Rahman M.M., Masum G.Z.H., Sharkar P., Sima S.N. (2013). Medicinal plant usage by traditional medical practitioners of rural villages in Chuadanga district, Bangladesh. Int. J. Biodivers. Sci. Ecost. Serv. Manag..

[159] Omar O.A.S., Bukhari H.M., ElSawy N.A., Header E.A. (2013). Efficacy of capsicum frutescens in curing the peptic ulcer. Int. J. Pure Appl. Sci. Technol..

[160] Mendivil E.J., Sandoval-Rodriguez A., Meza-Ríos A., Zuñiga-Ramos L., Dominguez-Rosales A., Vazquez-Del Mercado M., Sanchez-Orozco L., Santos-Garcia A., Armendariz-Borunda J. (2019). Capsaicin induces a protective effect on gastric mucosa along with decreased expression of inflammatory molecules in a gastritis model. J. Func. Foods.

[161] Santos P.L., Santos L.N.S., Ventura S.P.M., de Souza R.L., Coutinho J.A.P., Soares C.M.F., Lima Á.S. (2016). Recovery of capsaicin from Capsicum frutescens by applying aqueous two-phase systems based on acetonitrile and cholinium-based ionic liquids. Chem. Eng. Res. Des..

[162] Macalood J.S., Vicente H.J., Boniao R.D., Gorospe J.G., Roa E.C. (2013). Chemical analysis of *Carica papaya* L. Crude latex. Am. J. Plant. Sci..

[163] Nakhate Y.D., Talekar K.S., Giri S.V., Vasekar R.D., Mankar H.C., Tiwari P.R. (2019). Pharmacological and chemical composition of Carica papaya: On overview. World J. Pharm. Res..

[164] Santana L.F., Inada A.C., Santo E., Filiú W.F., Pott A., Alves F.M., Guimarães R.d.C.A., Freitas K.d.C., Hiane P.A. (2019). Nutraceutical potential of Carica papaya in metabolic syndrome. Nutrients.

[165] Vij T., Prashar Y. (2015). A review on medicinal properties of Carica papaya Linn. Asian Pac. J. Trop. Dis..

[166] Rahmani A.H., Aldebasi Y.H. (2016). Potential role of carica papaya and their active constituents in the prevention and treatment of diseases. Int. J. Pharm. Pharm. Sci..

[167] Kaur M., Talniya N.C., Sahrawat S., Kumar A., Stashenko E.E. (2019). Ethnomedicinal Uses, Phytochemistry and Pharmacology of Carica papaya Plant: A Compendious Review. Mini Rev. Org. Chem..

[168] Zhang C., Fan L., Fan S., Wang J., Luo T., Tang Y., Chen Z., Yu L. (2019). Cinnamomum cassia Presl: A review of its traditional uses, phytochemistry, pharmacology and toxicology. Molecules.

[169] Rao P.V., Gan S.H. (2014). Cinnamon: A multifaceted medicinal plant. Evid. Based Complement. Altern. Med..

[170] Gupta C., Kumari A., Garg A.P., Catanzaro R., Marotta F. (2011). Comparative study of cinnamon oil and clove oil on some oral microbiota. Acta Biomed..

[171] Muhammad J.S., Zaidi S.F., Shaharyar S., Refaat A., Usmanghani K., Saiki I., Sugiyama T. (2015). Anti-inflammatory effect of cinnamaldehyde in Helicobacter pylori induced gastric inflammation. Biol. Pharm. Bull..

[172] Vijayaraghavan K., Rajkumar J., Bukhari S.N.A., Al-Sayed B., Seyed M.A. (2017). Chromolaena odorata: A neglected weed with a wide spectrum of pharmacological activities. Mol. Med. Rep..

[173] Dulla O., Jahan F.I. (2017). Ethnopharmacological survey on traditional medicinal plants at Kalaroa Upazila, Satkhira district, Khulna Division, Bangladesh. J. Intercul. Ethnopharmacol..

[174] Paul T.S., Das B.B., Ingale S.P., Killedar N., Apte K.G. (2018). Oral intake of polyphenols of Chromolaena odorata: A perspective in peptic ulcer, thrombocytopenia, and heparin-induced bleeding diathesis in rodent model. Pharm. Res..

[175] Verma R.K., Kumari P., Maurya R.K., Kumar V., Verma R., Singh R.K. (2018). Medicinal properties of turmeric (*Curcuma longa* L.): A review. Int. J. Chem. Stud..

[176] Li S., Yuan W., Deng G., Wang P., Yang P., Aggarwal B. (2011). Chemical composition and product quality control of turmeric (*Curcuma longa* L.). Pharm. Crop..

[177] Ayati Z., Ramezani M., Amiri M.S., Moghadam A.T., Rahimi H., Abdollahzade A., Sahebkar A., Emami S.A. (2019). Ethnobotany, phytochemistry and traditional uses of Curcuma spp. and pharmacological profile of two important species (*C. longa* and *C. zedoaria*): A review. Curr. Pharm. Des..

[178] Rajkumari S., Sanatombi K. (2017). Nutritional value, phytochemical composition, and biological activities of edible Curcuma species: A review. Int. J. Food Prop..

[179] Omosa L., Midiwo J., Kuete V. (2017). Curcuma longa. Medicinal Spices and Vegetables from Africa.

[180] Rahmani A.H., Alsahli M.A., Aly S.M., Khan M.A., Aldebasi Y.H. (2018). Role of curcumin in disease prevention and treatment. Adv. Biomed. Res..

[181] Bachmeier B.E., Killian P.H., Melchart D. (2018). The role of curcumin in prevention and management of metastatic disease. Int. J. Mol. Sci..

[182] Vallée A., Lecarpentier Y. (2020). Curcumin and Endometriosis. Int. J. Mol. Sci..

[183] He Y., Yue Y., Zheng X., Zhang K., Chen S., Du Z. (2015). Curcumin, inflammation, and chronic diseases: How are they linked?. Molecules.

[184] Arshad L., Haque M.A., Abbas Bukhari S.N., Jantan I. (2017). An overview of structure–activity relationship studies of curcumin analogs as antioxidant and anti-inflammatory agents. Future Med. Chem..

[185] Yang H., Du Z., Wang W., Song M., Sanidad K., Sukamtoh E., Zheng J., Tian L., Xiao H., Liu Z. (2017). Structure–activity relationship of curcumin: Role of the methoxy group in anti-inflammatory and anticolitis effects of curcumin. J. Agric. Food Chem..

[186] Teng C.-F., Yu C.-H., Chang H.-Y., Hsieh W.-C., Wu T.-H., Lin J.-H., Wu H.-C., Jeng L.-B., Su I.-J. (2019). Chemopreventive effect of phytosomal curcumin on hepatitis b virus-related hepatocellular carcinoma in a transgenic mouse model. Sci. Rep..

[187] Singh A.G., Kumar A., Tewari D.D. (2012). An ethnobotanical survey of medicinal plants used in Terai forest of western Nepal. J. Ethnobiol. Ethnomed..

[188] Razafindraibe M., Kuhlman A.R., Rabarison H., Rakotoarimanana V., Rajeriarison C., Rakotoarivelo N., Randrianarivony T., Rakotoarivony F., Ludovic R., Randrianasolo A. (2013). Medicinal plants used by women from Agnalazaha littoral forest (Southeastern Madagascar). J. Ethnobiol. Ethnomed..

[189] Anand P., Kunnumakkara A.B., Newman R.A., Aggarwal B.B. (2007). Bioavailability of curcumin: Problems and promises. Mol. Pharm..

[190] Yang R., Yuan B.-C., Ma Y.-S., Zhou S., Liu Y. (2017). The anti-inflammatory activity of licorice, a widely used Chinese herb. Pharm. Biol..

[191] Chowdhary Z., Alamgir A., Alauddin M., Islan M., Chakma K., Hogue M., Kabir M. (2008). Traditional knowledge related to medicinal and aromatic plants in tribal societies and the quantitative study of alkaloids in medicinal plants of the hill tracts in Bangladesh. Pharm. Mag..

[192] Jalilzadeh-Amin G., Najarnezhad V., Anassori E., Mostafavi M., Keshipour H. (2015). Antiulcer properties of *Glycyrrhiza glabra* L. extract on experimental models of gastric ulcer in mice. Iran. J. Pharm. Res..

[193] Duke J.A. (2002). Handbook of Medicinal Herbs.

[194] Zhu Z., Tao W., Li J., Guo S., Qian D., Shang E., Su S., Duan J.A. (2016). Rapid determination of flavonoids in licorice and comparison of three licorice species. J. Sep. Sci..

[195] Fukai T., Marumo A., Kaitou K., Kanda T., Terada S., Nomura T. (2002). Anti-Helicobacter pylori flavonoids from licorice extract. Life Sci..

[196] Wittschier N., Faller G., Hensel A. (2009). Aqueous extracts and polysaccharides from liquorice roots (*Glycyrrhiza glabra* L.) inhibit adhesion of Helicobacter pylori to human gastric mucosa. J. Ethnopharmacol..

[197] Qu Y., Zong L., Xu M., Dong Y., Lu L. (2015). Effects of 18α-glycyrrhizin on TGF-β1/Smad signaling pathway in rats with carbon tetrachloride-induced liver fibrosis. Int. J. Clin. Exp. Pathol..

[198] Chatterjee A., Bandyopadhyay S.K. (2014). Herbal remedy: An alternate therapy of nonsteroidal anti-Inflammatory drug induced gastric ulcer healing. Ulcers.

[199] Zargari-Samadnejad A., Mehrvarz S., Allizadeh-Naeini S., Tanideh N. (2012). Healing effect of Licorice extract in acetic acid-induced ulcerative colitis in rat. Res. Pharm. Sci..

[200] Al-Snafi A.E. (2018). Chemical constituents, pharmacological effects and therapeutic importance of Hibiscus rosa-sinensis—A review. J. Pharm..

[201] Phani Kumar K., Annapurna A., Ramya G., Sheba D. (2014). Gastroprotective effect of flower extracts of Hibiscus rosa sinensis against acute gastric lesion models in rodents. J. Pharm. Phytochem..

[202] Sharma D., Bhatt S. (2014). Comprehensive review on ulcer healing potential of medicinal plants. Int. J. Pharm. Pharm. Sci..

[203] Bhaskar A., Nithya V. (2012). Evaluation of the wound-healing activity of *Hibiscus rosa sinensis* L. (Malvaceae) in Wistar albino rats. Indian J. Pharm..

[204] Harun N.H., Septama A.W., Ahmad W., Nizam W.A., Suppian R. (2019). The Potential of Centella asiatica (Linn.) Urban as an Anti-Microbial and Immunomodulator Agent: A Review. Nat. Prod. Sci..

[205] Sun B., Wu L., Wu Y., Zhang C., Qin L., Hayashi M., Kudo M., Gao M., Liu T. (2020). Therapeutic potential of Centella asiatica and its triterpenes: A review. Front. Pharm..

[206] Kasote D., Ahmad A., Viljoen A. (2015). Proangiogenic potential of medicinal plants in wound healing. Evidence-Based Validation of Herbal Medicine.

[207] Cheng C.L., Guo J.S., Luk J., Koo M.W.L. (2004). The healing effects of Centella extract and asiaticoside on acetic acid induced gastric ulcers in rats. Life Sci..

[208] Abdulla M., Al-Bayaty F., Younis L., Abu Hassan M. (2010). Anti-ulcer activity of Centella asiatica leaf extract against ethanol-induced gastric mucosal injury in rats. J. Med. Plant. Res..

[209] Bhowmik R., Saha M.R., Rahman M.A., Islam M.A.U. (2014). Ethnomedicinal survey of plants in the Southern District Noakhali, Bangladesh. Bangladesh Pharm. J..

[210] Mokganya M., Tshisikhawe M. (2019). Medicinal uses of selected wild edible vegetables consumed by Vhavenda of the Vhembe District Municipality, South Africa. S. Afr. J. Bot..

[211] Shah B., Seth A., Desai R. (2010). Phytopharmacological profile of Lagenaria siceraria: A review. Asian J. Plant. Sci..

[212] Manchala P. (2019). Evaluation of Anti-ulcer activity of Lagenaria siceraria chloroform extracts in pylorus ligated rats. Electron. J. Biol..

[213] Shendge P.N., Belemkar S. (2019). Acute and 28-day oral toxicity studies of methanolic extract of *Lagenaria siceraria* (Cucurbitaceae) fruit in rats. Drug Chem. Toxicol..

[214] Batool N., Ilyas N., Shabir S., Saeed M., Mazhar R., Amjid M.W. (2018). A mini-review of therapeutic potential of *Mangifera indica* L.. Pak. J. Pharm. Sci..

[215] Neelima N., Sudhakar M., Patil M.B., Lakshmi B. (2012). Anti-ulcer activity and HPTLC analysis of *Mangifera indica* L. leaves. Int. J. Pharm. Phytopharm. Res..

[216] Tumpa S.I., Hossain M.I., Ishika T. (2014). Ethnomedicinal uses of herbs by indigenous medicine practitioners of Jhenaidah district, Bangladesh. J. Pharm. Phytochem..

[217] Stohs S., Swaroop A., Moriyama H., Bagchi M., Ahmad T., Bagchi D. (2018). A Review on Antioxidant, Anti-Inflammatory and Gastroprotective Abilities of Mango (*Magnifera indica*) Leaf Extract and Mangiferin. J. Nutr. Health Sci..

[218] Chand R., Devi S., Seeristi S., Kumar S., Goundar N., Naranyan N., Chandra P. (2018). Traditional use of Medicinal plants among selected Villages in Fiji Islands: A Review Perspective. Pac. Med. Stud. J..

[219] Rahman A. (2015). Ethnomedicinal survey of angiosperm plants used by Santal tribe of Joypurhat District, Bangladesh. Int. J. Adv. Res. Dev..

[220] Vinothapooshan G., Sundar K. (2010). Anti-ulcer activity of Mimosa pudica leaves against gastric ulcer in rats. Res. J. Pharm. Biol. Chem. Sci..

[221] Zaware B., Gilhotra R., Chaudhari S.R. (2018). Potential of Mimosa pudica leaf in the treatment of ulcerative colitis in rat. Bangladesh J. Pharm..

[222] Shahadat H., Mostofa M., Mamum M., Hoque M., Awal M. (2008). Comparative efficacy of korolla (Momordica charantia) extract and IvermecÂ^®^ pour on with their effects on certain blood parameters and body weight gain in indigenous chicken infected with Ascaridia galli. Bangladesh J. Vet. Med..

[223] Rahmatullah M., Khairuzzaman M., Saleem S.M., Sattar F., Rahman I.l., Yesmin M.S., Malek I., Bashar A.B.M.A. (2015). Documentation of some folk medicinal practices in Sylhet &Moulavibazar districts, Bangladesh. World J. Pharm. Pharm. Sci..

[224] Gill N., Rani P., Arora R., Dhawan V., Bali M. (2012). Evaluation of antioxidant, antiinflammatory and antiulcer potential of Momordica charantia methanolic seed extract. Res. J. Phytochem..

[225] Shahrokhi N., Keshavarzi Z., Khaksari M. (2015). Ulcer healing activity of Mumijo aqueous extract against acetic acid induced gastric ulcer in rats. J. Pharm. Bioallied Sci..

[226] Alam S., Asad M., Asdaq S.M.B., Prasad V.S. (2009). Antiulcer activity of methanolic extract of *Momordica charantia* L. in rats. J. Ethnopharmacol..

[227] Rajendran A., Sureshkumar S. (2019). Phytonutrients: Moringa oleifera leaf extracts anincredible health super food supplement. Pharm. Innov. J..

[228] Vimala G., Gricilda Shoba F. (2014). A review on antiulcer activity of few Indian medicinal plants. Int. J. Microbiol..

[229] Kleemann R., Verschuren L., Morrison M., Zadelaar S., van Erk M.J., Wielinga P.Y., Kooistra T. (2011). Anti-inflammatory, anti-proliferative and anti-atherosclerotic effects of quercetin in human in vitro and in vivo models. Atherosclerosis.

[230] Choudhary M.K., Bodakhe S.H., Gupta S.K. (2013). Assessment of the antiulcer potential of Moringa oleifera root-bark extract in rats. J. Acupunct. Meridian Stud..

[231] Devaraj V., Asad M., Prasad S. (2007). Effect of Leaves and Fruits of Moringa oleifera. on Gastric and Duodenal Ulcers. Pharm. Biol..

[232] Parvez G.M., Shakib U., Khokon M., Sanzia M. (2018). A short review on a nutritional fruit: Guava. Toxicol. Res..

[233] Rahman A., Kumar A. (2015). Investigation of medicinal plants at Katakhali Pouroshova of Rajshahi District, Bangladesh and their conservation management. Appl. Ecol. Environ. Sci..

[234] Tuhin M.I.H., Asaduzzaman M., Islam E., Khatun Z., Rahmatullah M. (2013). Medicinal plants used by folk medicinal herbalists in seven villages of Bhola district, Bangladesh. Am. Eur. J. Sustain. Agric..

[235] Choudhary A., Singh A., Duggal N., Kumar B. (2014). Fruits of Scindapsus officinalis attenuates pylorus ligation induced ulcer in rats. Int. J. Pharm. Sci. Res..

[236] Darekar S.M., Jayakumari S. (2018). A review on wound healing activity of different extracts and formulations of Shorea robusta resin. Drug Invent. Today.

[237] Santhoshkumar M., Anusuya N., Bhuvaneswari P. (2012). Antiulcerogenic effect of resin from Shorea robusta Gaertn. f. on experimentally induced ulcer models. Int. J. Pharm. Pharm. Sci.

[238] Eskandari M., Assadi M., Shirzadian S., Mehregan I. (2019). Ethnobotanical Study and Distribution of the Solanum Section Solanum Species (Solanaceae) in Iran. J. Med. Plant..

[239] Mayilsamy M., Rajendran A. (2013). Ethnomedicinal plants used by paliyar tribals in Dindigul district of Tamil Nadu, India. Int. J. Sci. Innov. Dis..

[240] Mittal M., Gupta N., Parashar P., Mehra V., Khatri M. (2014). Phytochemical evaluation and pharmacological activity of Syzygium aromaticum: A comprehensive review. Int. J. Pharm. Pharm. Sci..

[241] Bhowmik D., Kumar K.S., Yadav A., Srivastava S., Paswan S., Dutta A.S. (2012). Recent trends in Indian traditional herbs Syzygium aromaticum and its health benefits. J. Pharm. Phytochem..

[242] Winnie G.M. (2018). A study on medicinal plants used in ‘Karkidaka Kanji’: The ayurvedic medicine. Int. J. Sci. Res. Sci. Eng. Technol..

[243] Batiha G.E.-S., Alkazmi L.M., Wasef L.G., Beshbishy A.M., Nadwa E.H., Rashwan E.K. (2020). *Syzygium aromaticum* L. (Myrtaceae): Traditional uses, bioactive chemical constituents, pharmacological and toxicological activities. Biomolecules.

[244] Shaheen E.K., Syef A., Saha S.S., Islam S., Al Hossain D., Sujan A.I., Rahmatullah M. (2011). Medicinal plants used by the folk and tribal medicinal practitioners in two villages of Khakiachora and Khasia Palli in Sylhet district, Bangladesh. Adv. Nat. Appl. Sci..

[245] Roy S., Uddin M.Z., Hassan M.A., Rahman M.M. (2008). Medico-botanical report on the Chakma community of Bangladesh. Bangladesh J. Plant. Taxon..

[246] Mishra V., Agrawal M., Onasanwo S.A., Madhur G., Rastogi P., Pandey H.P., Palit G., Narender T. (2013). Anti-secretory and cyto-protective effects of chebulinic acid isolated from the fruits of Terminalia chebula on gastric ulcers. Phytomedicine.

[247] Saha S., Ghosh S. (2012). *Tinospora cordifolia*: One plant, many roles. Anc. Sci. Life.

[248] Kaur M., Singh A., Kumar B. (2014). Comparative antidiarrheal and antiulcer effect of the aqueous and ethanolic stem bark extracts of *Tinospora cordifolia* in rats. J. Adv. Pharm. Technol. Res..

[249] Upadhyay A.K., Kumar K., Kumar A., Mishra H.S. (2010). *Tinospora cordifolia* (Willd.) Hook. f. and Thoms.(Guduchi)–validation of the Ayurvedic pharmacology through experimental and clinical studies. Int. J. Ayurveda Res..

[250] Toda K., Hitoe S., Takeda S., Shimoda H. (2016). Black ginger extract increases physical fitness performance and muscular endurance by improving inflammation and energy metabolism. Heliyon.

[251] Shahrajabian M.H., Sun W., Cheng Q. (2019). Clinical aspects and health benefits of ginger (*Zingiber officinale*) in both traditional Chinese medicine and modern industry. Acta Agric. Scand. B Soil Plant. Sci..

[252] Asnaashari S., Dastmalchi S., Javadzadeh Y. (2018). Gastroprotective effects of herbal medicines (roots). Int. J. Food Prop..

[253] Al-Howiriny T., Alsheikh A., Alqasoumi S., Al-Yahya M., ElTahir K., Rafatullah S. (2010). Gastric antiulcer, antisecretory and cytoprotective properties of celery (*Apium graveolens*) in rats. Pharm. Biol..

[254] Singh D.P., Borse S.P., Nivsarkar M. (2016). A novel model for NSAID induced gastroenteropathy in rats. J. Pharm. Toxicol. Methods.

[255] Ahmad B., Rehman M.U., Amin I., Arif A., Rasool S., Bhat S.A., Afzal I., Hussain I., Bilal S. (2015). A review on pharmacological properties of zingerone (4-(4-Hydroxy-3-methoxyphenyl)-2-butanone). Sci. World J..

[256] Sistani Karampour N., Arzi A., Rezaie A., Pashmforoosh M., Kordi F. (2019). Gastroprotective effect of zingerone on ethanol-induced gastric ulcers in rats. Medicina.

[257] Zhu Y., Wang F., Zhao Y., Wang P., Sang S. (2017). Gastroprotective [6]-gingerol aspirinate as a novel chemopreventive prodrug of aspirin for colon cancer. Sci. Rep..

[258] Khedr A. (2017). Antiulcer protective activity of gum Arabic (Acacia Senegal) in adult rats. Bull. Natl. Nutr. Inst. Arab Repub. Egypt.

[259] Sohel M., Kawsar M., Sumon M., Sultana T. (2016). Ethnomedicinal studies of Lalmohan Thana in Bhola district, Bangladesh. Altern. Integr. Med..

[260] Azam M.N.K., Ahmed M.N., Rahman M.M., Rahmatullah M. (2013). Ethnomedicines used by the Oraon and Gor tribes of Sylhet district, Bangladesh. Am. Eur. J. Sustain. Agric..

[261] Maury P.K., Jain S., Lal N., Alok S. (2012). A review on antiulcer activity. Int. J. Pharm. Sci. Res..

[262] Dey A. (2011). *Achyranthes aspera* L: Phytochemical and pharmacological aspects. Int. J. Pharm. Sci. Rev. Res..

[263] Agrawal T. (2018). Allophylus serratus; A pharmacologically important plant. World J. Pharm. Res..

[264] Vanita K., Deepali M. (2019). Evaluation of antipyretic and antiulcer activity of ethanolic extract of leaves of *Alstonia scholaris l*. In albino wistar rats. Asian J. Pharm. Clin. Res..

[265] Reyad-ul-Ferdous M., Shamim Shahjahan D., Sharif T., Mohsina M. (2015). Present biological status of potential medicinal plant of amaranthus viridis: A comprehensive review. Am. J. Clin. Exp. Med..

[266] Wong V., Yu L., Cho C. (2008). Protective effect of polysaccharides from Angelica sinensis on ulcerative colitis in rats. Inflammopharmacology.

[267] Rahman M.S., Rahman M.Z., Uddin A.A., Rashid M.A. (2007). Steriod and Triterpenoid from Anogeissus latifolia. Dhaka Univ. J. Pharm. Sci..

[268] Veitch G.E., Beckmann E., Burke B.J., Boyer A., Maslen S.L., Ley S.V. (2007). Synthesis of azadirachtin: A long but successful journey. Angew. Chem. Int. Ed..

[269] Anbari K., Hasanvand A., Andevari A.N., Abbaszadeh S. (2019). Concise overview: A review on natural antioxidants and important herbal plants on gastrointestinal System. Res. J. Pharm. Technol..

[270] Azad A., Mahmud M.R., Parvin A., Chakrabortty A., Akter F., Moury S.I., Anny I.P., Rehnoma S., Tarannom S.K.J., Chowdhury S.Y. (2014). Medicinal plants of a folk medicinal healer of Rangpur district, Bangladesh. J. Med. Plants.

[271] Singh A., Rani R., Sharma M. (2018). Medicinal herbs of Punjab (India). Int. J..

[272] Deng R. (2007). Therapeutic effects of guggul and its constituent guggulsterone: Cardiovascular benefits. Cardiovasc. Drug Rev..

[273] Al-Snafi A.E. (2018). Arabian medicinal plants possessed gastroprotective effects-plant based review (part 1). IOSR J. Pharm..

[274] Ahmed M., Azam K., Nur M. (2014). Traditional knowledge and formulations of medicinal plants used by the traditional medical practitioners of Bangladesh to treat schizophrenia like psychosis. Schizophr. Res. Treat..

[275] Arefin M., Hossain M., Hossain M.A. (2017). Plant diversity of Sonadia Island An ecologically critical area of South-East Bangladesh. Bangladesh J. Plant. Taxon..

[276] Gregory M., Divya B., Mary R.A., Viji M.H., Kalaichelvan V., Palanivel V. (2013). Anti–ulcer activity of Ficus religiosa leaf ethanolic extract. Asian Pac. J. Trop. Biomed..

[277] Rahman A., Khanom A. (2013). Taxonomic and Ethno-Medicinal Study of Species from Moraceae (Mulberry) Family in Bangladesh Flora. Res. Plant. Sci..

[278] Zhou Y.-H., Yu J.-P., Liu Y.-F., Teng X.-J., Ming M., Lv P., An P., Liu S.-Q., Yu H.-G. (2006). Effects of Ginkgo biloba extract on inflammatory mediators (SOD, MDA, TNF-α, NF-κBp65, IL-6) in TNBS-induced colitis in rats. Mediat. Inflamm..

[279] Rahman A.M., Akter M. (2015). Taxonomy and traditional medicinal uses of Apocynaceae (Dogbane) family of Rajshahi district, Bangladesh. Res. Rev. J. Bot. Sci..

[280] Poornima N., Umarajan K., Babu K. (2009). Studies on anatomical and phytochemical analysis of Oxystelma esculentum (Lf) R. br. Ex Schltes. Bot. Res. Int..

[281] Urmistha S., Ankit S., Mrityunjoy M. (2019). Anti-ulcer activity of hydroalcoholic extract of *Piper betle* leaf on experimental animals. Asian J. Pharm. Clin. Res..

[282] Tanna A., Nair R., Chanda S. (2009). Assessment of anti-inflammatory and hepatoprotective potency of Polyalthia longifolia var. pendula leaf in Wistar albino rats. J. Nat. Med..

[283] Mahmud S., Mahmud S., Hasan M.K., Rahman S., Kar A., Shathy E.J., Mohiuddin A. (2016). A survey on medicinal plants usage by folk medicinal practitioners in different villages of Jhenaigati Upazila, Sherpur district, Bangladesh. J. Pharm. Phytochem..

[284] Bhalke R.D., Giri M.A., Anarthe S.J., Pal S.C. (2010). Antiulcer activity of the ethanol extract of leaves of *Sesbania grandiflora* (Linn.). Int. J. Pharm. Pharm. Sci..

[285] Uddin M.Z., Hassan M.A. (2016). Plant diversity of Dhaka university campus, Bangladesh. J. Asiat. Soc. Bangladesh Sci..

[286] Sen S., Chakraborty R., Debnath B. (2011). Challenges and opportunities in the advancement of herbal medicine: India’s position and role in a global context. J. Herb. Med..

